# Bimetallic Aluminum 5,6-Dihydro-7,7-dimethyl quinolin-8-olates as Pro-Initiators for the ROP of ε-CL; Probing the Nuclearity of the Active Initiator

**DOI:** 10.3390/polym10070764

**Published:** 2018-07-12

**Authors:** Qiurui Zhang, Wenjuan Zhang, Gregory A. Solan, Tongling Liang, Wen-Hua Sun

**Affiliations:** 1 Beijing Key Laboratory of Clothing Materials R&D and Assessment, Beijing Engineering Research Center of Textile Nanofiber, School of Materials Science and Engineering, Beijing Institute of Fashion Technology, Beijing 100029, China; zhangqiurui@iccas.ac.cn; 2 Key Laboratory of Engineering Plastics and Beijing National Laboratory for Molecular Science, Institute of Chemistry, Chinese Academy of Sciences, Beijing 100190, China; ltl@iccas.ac.cn; 3 Department of Chemistry, University of Leicester, University Road, Leicester LE1 7RH, UK

**Keywords:** aluminum complexes, ring opening polymerization, ε-caprolactone, bidentate, reaction mechanism

## Abstract

Six examples of aluminum 5,6-dihydro-7,7-dimethylquinolin-8-olates, [{2-R^1^-7,7-Me_2_-8-R^2^C_9_H_6_N-8-O}AlR^3^_2_]_2_ (R^1^ = R^2^ = H, R^3^ = Me **C1**; R^1^ = R^2^ = H, R^3^ = Et **C2**; R^1^ = R^2^ = H, R^3^ = *i*-Bu **C3**; R^1^ = Cl, R^2^ = H, R^3^ = Me **C4**; R^1^ = H, R^2^ = R^3^ = Me **C5**; R^1^ = Cl, R^2^ = R^3^ = Me **C6**), have been prepared by treating the corresponding pro-ligand (**L1**–**L4**) with either AlMe_3_, AlEt_3_ or Al(*i*-Bu)_3_. All complexes have been characterized by ^1^H and ^13^C NMR spectroscopy and in the case of **C1** and **C4** by single crystal X-ray diffraction; dimeric species are a feature of their molecular structures. In the presence of PhCH_2_OH (BnOH), **C1**–**C6** displayed good control and efficiency for the ROP of ε-CL with almost 100% conversion achievable in 10 min at 90 °C; the chloro-substituted **C4** and **C6** notably exhibited the lowest activity of the series. However, in the absence of BnOH, **C1** showed only low activity with 15% conversion achieved in 30 min forming a linear polymer capped with either a methyl or a **L1** group. By contrast, when one or more equivalents of BnOH was employed in combination with **C1**, the resulting catalyst was not only more active but gave linear polymers capped with BnO end-groups. By using ^1^H and ^27^Al NMR spectroscopy to monitor solutions of **C1**, **C1**/BnOH and **C1/**BnOH/10 ε-CL over a range of temperatures, some support for a monomeric species being the active initiator at the operational temperature is presented.

## 1. Introduction

The past 10 years or so have seen some rapid progress in the synthesis of biodegradable polymers and in particular, aliphatic polyesters such as polylactides (PLA) and poly(ε-caprolactone) (PCL). These developments can be attributed, in a large measure, to the good biodegradability and biocompatibility properties of these materials as well as to their ease of preparation [1,2,3,4]. Typically, such polyesters can be prepared by the ring-opening polymerization (ROP) of cyclic esters catalyzed by metal complexes such as those based on Al, Ca, Sn and rare earth metals. In addition, immobilized catalysts have been considered for the improvement of the mechanical properties of PCLs [5]. Several review articles have documented advances in catalyst design for the ROP of cyclic esters [6,7]. Among the numerous reports, aluminum complexes bearing multidentate ligands such as Salen-Al and Salan-Al have attracted much attention due to their relatively high Lewis acidity, good controllability, as well as their decent selectivity towards the ROP of *rac*-lactide (*rac*-LA) and ε-caprolactone (ε-CL) [8,9,10,11]. By way of contrast, there are still relatively few examples of bidentate N^O-type aluminum complexes that have been used effectively for the ROP of cyclic esters.

**Chart 1:**
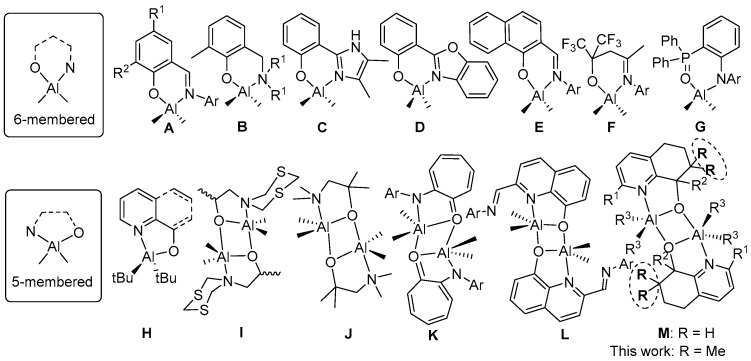
Reported N^O-bidentate aluminum dialkyl complexes.

With regard to N^O-aluminum complexes containing six-membered chelate rings (**A**–**G**, Chart 1), the 2-iminophenolates **A** constitute the most studied class of pro-initiator and indeed are highly effective in the presence of benzyl alcohol for the ROP of ε-CL and LA [12,13,14,15]. They can also efficiently promote the copolymerization of *rac*-lactide and glycolide [16], *rac*-β-butyrolactone and *L*-lactide [17] and the random copolymerization of *rac*-LA and ε-CL with various degrees of control [18]. Related bimetallic aluminum complexes incorporating two linked iminophenolate units have also been evaluated in the ROP of cyclic esters [19]. The dialkylaluminum aminophenolate complexes **B** have been shown as effective (pro-)initiators for the ROP of ε-CL and epoxides [20,21,22,23], while their dinuclear analogues showed higher activity [22,23]; good selectivity for the ROP of *rac*-LA have also been noted for some **B**-type systems [24]. Other aluminum phenolates such as dialkylaluminum 2-imidazolylphenolates (**C**) and 2-benzoxazole phenolates (**D**) have also been reported as efficient (pro-)initiators for the ROP of *rac*-lactide and ε-CL [25,26]. The β-ketiminato-aluminum complex **E** showed good efficiency for the random copolymerization of LA and ε-CL [18,27], while the fluorinated alkoxy-imino aluminum species **F** are active for the ROP of ε-CL [28]. Conversely, the amido-phosphinoxide aluminum complex **G** showed virtually no activity for the ROP of ε-CL [29].

In comparison with the six-membered ring examples highlighted above (**A**–**G**, Chart 1), those based on five-membered N^O-chelate rings have been considerably less studied as ROP (pro-)initiators, though a tendency to form dimeric species is a feature of their structural chemistry (**H**–**M**, Chart 1). Nevertheless, a recent report has shown that five-membered ring aluminum complexes can display significantly higher polymerization rates when compared to their six-membered ring counterparts (e.g., two to three-fold increase for ε-CL polymerization) [30]. Elsewhere, N^O-bidentate aluminum complexes of type **H** [31,32] and **J** have been reported but have not been the subject of ROP studies though **H** has been discussed in terms of the relative stability of its mono- and dimeric forms [33]. On the other hand, dimeric **J** was shown to catalyze the cycloaddition of CO_2_ with epoxides [34]. Moreover, the anilinotropone-based dimeric aluminum complexes **K** exhibited, in the presence of BnOH, high activity in the ROP of *rac*-lactide [35]. Nonetheless, common to **J** and **K** it remains uncertain whether the dimeric structural forms are maintained during the polymerization or undergo dissociation to their monomeric forms. Interestingly, we have found the di- and monomeric complexes of dialkylaluminum 2-(arylimino)-quinolin-8-olates (**L**) display very different catalytic performance for the ROP of ε-CL. For example, the binuclear systems showed very low activity, while the mononuclear examples gave much higher efficiency and in a more controlled manner [36]. Furthermore, our group has recently synthesized a series of bimetallic dialkylaluminum hydroquinolin-8-olates (**M**: R^1^ = H, Chart 1) that showed good activity only at high temperature for the ROP of ε-CL. By using variable temperature ^1^H and ^27^Al NMR spectroscopy, we proposed that the di-aluminum complexes partly dissociated into mononuclear species at high temperature [37]. However, these complexes displayed only poor solubility at room temperature which precluded full assignments of their peaks in their ^1^H NMR spectra.

With a view to improve the solubility of **M** (Chart 1) and to re-investigate the nuclearity of the active initiator, we report herein the introduction of two methyl groups at the 7-position of the ligand framework. In particular, we report a series of bimetallic aluminum 5,6-dihydro-7,7-dimethylquinolin-8-olates, [{2-R^1^-7,7-Me_2_-8-R^2^C_9_H_6_N-8-O}AlR^3^_2_]_2_ (R^1^ = R^2^ = H, R^3^ = Me; R^1^ = R^2^ = H, R^3^ = Et; R^1^ = R^2^ = H, R^3^ = *i*-Bu; R^1^ = Cl, R^2^ = H, R^3^ = Me; R^1^ = H, R^2^ = R^3^ = Me; R^1^ = Cl, R^2^ = R^3^ = Me), that differ in the substitution pattern at the R^1^, R^2^ and R^3^ positions. A full evaluation of these complexes as either initiators or pro-initiators for the ROP of ε-CL is conducted and these results compared with those observed using parent **M**. In addition, the pathway by which pro-initiator is transformed in to the initiator is probed using both ^1^H and ^27^Al NMR spectroscopy.

## 2. Materials and Methods

### 2.1. General Considerations and Materials

All manipulations of air or moisture-sensitive compounds were performed using standard Schlenk techniques under an atmosphere of high-purity nitrogen or using glove box techniques. Toluene was dried by refluxing it over sodium/benzophenone and distilled under nitrogen and stored over activated molecular sieves (4 Å) for 24 h in a glove box prior to use. *n*-Hexane and CDCl_3_ were dried over CaH_2_ for 48 h, distilled under nitrogen and stored over activated molecular sieves (4 Å) in a glove box prior to use. Solutions of Me_3_Al (1.0 M in toluene), Et_3_Al (1.0 M in toluene), *i*-Bu_3_Al (1.0 M in toluene) and methyllithium (1.0 M in toluene) were purchased from Aldrich and used as received. Elemental analyses were performed using a PE2400II Series instrument (Perkin-Elmer Co., Shanghai, China). The ^1^H and ^13^C NMR spectra were recorded on a Bruker DMX-400/300 MHz (Karlsruhe, Germany) spectrometer using TMS as an internal standard; δ values are given in ppm and *J* values in Hz. The ^27^Al NMR spectra were recorded on Bruker 500 MHZ (Beijing, China) spectrometer. The NMR spectra of the complexes and ligands were recorded in CDCl_3_ at room temperature. IR spectra were recorded on a Perkin-Elmer System 2000 (Shanghai, China) FT-IR spectrometer. The GPC measurements were performed using a set-up based on a Waters-1515 HPLC pump, a Waters 2414 (Beijng, China) refractive index detector and a combination of Styragel HT-2, HT-3 and HT-4 columns, the effective molar mass ranges of which are 100–10,000, 500–30,000 and 5000–600,000, respectively. THF was used as the eluent (flow rate: 1 mL/min, at 35 °C). Molecular weights and molecular weight distributions were calculated using polystyrene as a standard.

### 2.2. Syntheses of 2-R^1^-7,7-Me_2_,8-R^2^C_9_H_6_N-8-OH (L)

R^1^ = R^2^ = H **L1**. A mixture of 5,6-dihydro-7,7-dimethylquinolin-8-one (0.88 g, 5.0 mmol) and sodium borohydride (0.19 g, 5.0 mmol) was dissolved in methanol (30 mL) in a 100 mL round flask and stirred for 5 h at room temperature. The reaction was then quenched with water, extracted with dichloromethane and the organic phase dried over anhydrous magnesium sulfate and filtered. The filtrate was collected and the solvent evaporated under reduced pressure to give **L1** as a light yellow solid. Yield: 0.86 g (96%). ^1^H NMR (400 MHz, CDCl_3_, ppm): δ 8.40 (d, *J* = 6.0 Hz, 1H, Py–*H*), 7.41 (d, *J* = 8.0 Hz, 1H, Py–*H*), 7.14–7.11 (m, 1H, Py–*H*), 4.38 (s, 1H, C*H*–OH), 4.31 (s, 1H, O*H*), 2.92–2.79 (m, 2H, C*H*_2_), 1.75–1.65 (m, 2H, C*H*_2_), 1.19 (s, 3H, C*H*_3_), 0.85 (s, 3H, C*H*_3_). ^13^C NMR (100 MHz, CDCl_3_, ppm): δ 157.3, 146.3, 136.5, 130.4, 122.0, 76.2, 34.0, 33.3, 27.5, 24.8, 18.9. IR (cm^−1^): 3117 (m), 2953 (m), 2922 (m), 2859 (w), 2714 (w), 1583 (m), 1446 (m), 1377 (m), 1359 (m), 1307 (w), 1260 (m), 1160 (m), 1107 (m), 1042 (s), 981 (m), 936 (w), 904 (w), 857 (w), 788 (s), 751 (m), 713 (m). Anal. Calcd for C_11_H_15_NO: C, 74.54; H, 8.53; N, 7.90%. Found: C, 74.40; H, 8.53; N, 7.81%.

R^1^ = Cl, R^2^ = H **L2**. Using a similar procedure to that described for **L1**, but with 2-chloro-5,6-dihydro-7,7-dimethylquinolin-8-one as the ketone, gave **L2** as a white powder. Yield: 1.03 g (97%). ^1^H NMR (400 MHz, CDCl_3_, ppm): δ 7.38 (d, *J* = 8.0 Hz, 1H, Py–*H*), 7.14 (d, *J* = 8.0 Hz, 1H, Py–*H*), 4.27 (s, 1H, CH–O*H*), 3.76 (s, 1H, O*H*), 2.85–2.69 (m, 2H, C*H*_2_), 1.71–1.67 (m, 2H, C*H*_2_), 1.16 (s, 3H, C*H*_3_), 0.87 (s, 3H, C*H*_3_). ^13^C NMR (100 MHz, CDCl_3_, ppm): *δ* 158.3, 148.6, 139.6, 129.0, 123.0, 76.0, 34.0, 33.3, 27.5, 24.3, 19.0. IR (cm^−1^): 3281 (m), 2943 (m), 2905 (m), 2865 (w), 1570 (m), 1469 (m), 1440 (m), 1416 (w), 1377 (m), 1363 (m), 1256 (m), 1230 (w), 1197 (w), 1164 (m), 1131 (w), 1090 (m), 1033 (s), 946 (m), 904 (w), 870 (w), 842 (m), 789 (s), 756 (m), 727 (m). Anal. Calcd for C_11_H_14_ClNO: C, 62.41; H, 6.67; N, 6.62%. Found: C, 62.58; H, 6.59; N, 6.43%.

R^1^ = H, R^2^ = Me **L3**. Methyllithium (5.0 mL, 5.0 mmol, 1.0 M solution in toluene) was added dropwise to a stirred solution of 5,6-dihydro-7,7-dimethylquinolin-8-one (0.88 g, 5.0 mmol) in toluene (10.0 mL) at −30 °C. The reaction mixture was allowed to warm slowly to room temperature and stirred overnight. Following quenching with water and extraction with dichloromethane, the organic phase was dried over anhydrous magnesium sulfate and filtered. The filtrate was collected and the solvent removed under reduced pressure to give **L3** as a white solid. Yield: 0.93 g (97%). ^1^H NMR (400 MHz, CDCl_3_, ppm): *δ* 8.39 (d, *J* = 4.0 Hz, 1H, Py–*H*), 7.38 (d, *J* = 8.0 Hz, 1H, Py–*H*), 7.11–7.08 (m, 1H, Py–*H*), 4.25 (s, 1H, O*H*), 2.90–2.74 (m, 2H, C*H*_2_), 1.94–1.86 (m, 1H, C*H*_2_), 1.63–1.58 (m, 1H, C*H*_2_), 1.37 (s, 1H, C*H*_3_), 1.15 (s, 1H, C*H*_3_), 0.93 (s, 1H, C*H*_3_). ^13^C NMR (100 MHz, CDCl_3_, ppm): *δ* 162.3, 148.3, 139.6, 128.2, 122.4, 74.7, 36.1, 33.0, 26.7, 24.5, 23.9, 21.9. IR (cm^−1^): 3413 (m), 2959 (m), 2872 (w), 1702 (w), 1580 (m), 1447 (m), 1424 (m), 1386 (m), 1359 (m), 1336 (w), 1283 (m), 1260 (m), 1169 (m), 1131 (m), 1077 (s), 1018 (m), 978 (m), 934 (w), 907 (w), 789 (s). Anal. Calcd for C_12_H_17_NO: C, 75.35; H, 8.96; N, 7.32%. Found: C, 75.26; H, 8.82; N, 7.46%.

R^1^ = Cl, R^2^ = Me **L4**. Using a similar procedure to that described for **L3**, but with 2-chloro-5,6-dihydro-7,7-dimethylquinolin-8-one as the ketone, gave **L4** as a white powder. Yield: 1.09 g (97%). ^1^H NMR (400 MHz, CDCl_3_, ppm): 7.34 (d, *J* = 8.0 Hz, 1H, Py–*H*), 7.11 (d, *J* = 8.0 Hz, 1H, Py–*H*), 3.65 (s, 1H, O*H*), 2.87–2.70 (m, 2H, C*H*_2_), 1.89–1.81 (m, 1H, C*H*_2_), 1.65–1.59 (m, 1H, C*H*_2_), 1.38 (s, 3H, C*H*_3_), 1.12 (s, 3H, C*H*_3_), 0.94 (s, 3H, C*H*_3_). ^13^C NMR (100 MHz, CDCl_3_, ppm): *δ* 161.4, 146.5, 136.5, 129.2, 121.8, 74.9, 36.2, 33.0, 27.1, 24.5, 24.4, 22.0. IR (cm^−1^): 3477 (m), 2963 (m), 2924 (m), 1697 (m), 1572 (m), 1444 (m), 1427 (m), 1385 (m), 1360 (m), 1328 (m), 1316 (w), 1261 (m), 1191 (w), 1166 (m), 1127 (m), 1069 (s), 1016 (m), 932 (w), 854 (w), 815 (s), 748 (m). Anal. Calcd for C_12_H_16_ClNO: C, 63.86; H, 7.15; N, 6.21%. Found: C, 63.45; H, 7.28; N, 6.46%.

### 2.3. Syntheses of [{2-R^1^-7,7-Me_2_-8-R^2^C_9_H_6_N-8-O}AlR_3_^2^]_2_ (C)

R^1^ = R^2^ = H, R^3^ = Me **C1.** Me_3_Al (5.0 mL, 5.0 mmol, 1.0 M solution in toluene) was added dropwise to a stirred solution of **L1** (0.89 g, 5.0 mmol) in toluene (10 mL) at −78 °C. The resulting solution was allowed to warm slowly to room temperature and stirred for 3 h. Following concentration of the reaction mixture to ca. 1 mL, hexane (10 mL) was added to induce precipitation. The precipitate was filtered affording **C1** as a white powder. Yield: 0.75 g, 65%. ^1^H NMR (400 MHz, toluene-*d*_8_, ppm): *δ* 7.44 (d, *J* = 8.0 Hz, 1H, Py–*H*), 6.63 (d, *J* = 8.0 Hz, 1H, Py–*H*), 6.47–6.41 (m, 1H, Py–*H*), 4.58 (s, 1H, C*H*–O), 2.07–1.98 (m, 2H, C*H*_2_), 1.52 (s, 3H, C*H*_3_), 1.21–1.13 (m, 2H, C*H*_2_), 0.68 (s, 3H, C*H*_3_), −0.12 (s, 3H, Al–C*H*_3_), −0.26 (s, 3H, Al–C*H*_3_). ^13^C NMR (100 MHz, toluene-*d*_8_, ppm): *δ* 157.0, 141.7, 140.1, 134.2, 124.0, 82.1, 36.2, 35.8, 30.1, 24.2, 17.7, −3.26. Anal. Calcd for C_13_H_20_AlNO: C, 66.93; H, 8.64; N, 6.00%. Found: C, 67.13; H, 8.77; N, 5.88%.

R^1^ = R^2^ = H, R^3^ = Et **C2**. Using a similar procedure to that described for **C1**, but with Et_3_Al (1.0 M solution in toluene) as the alkyl aluminum reagent, gave **C2** as a white powder. Yield: 0.55 g, 42%. ^1^H NMR (400 MHz, toluene-*d*_8_, ppm): δ 8.11 (d, *J* = 4.0 Hz, 1H, Py–*H*), 6.74 (d, *J* = 5.6 Hz, 1H, Py–*H*), 6.57 (t, *J* = 6.0 Hz, 1H, Py–*H*), 5.01 (s, 1H, C*H*–O), 2.34–2.22 (m, 2H, C*H*_2_), 1.54 (s, 3H, C*H*_3_), 1.45–1.37 (m, 1H, Al–CH_2_C*H*_3_), 1.30–1.25 (m, 2H, C*H*_2_), 1.21–1.15 (m, 3H, Al–CH_2_C*H*_3_), 0.94 (s, 3H, CH_3_ and Al–CH_2_C*H*_3_), 0.51–0.44 (m, 2H, Al–C*H*_2_CH_3_), 0.35–0.26 (m, 2H, Al–C*H*_2_CH_3_). ^13^C NMR (100 MHz, toluene-*d*_8_, ppm): *δ* 164.4, 158.5, 142.5, 132.1, 122.7, 80.9, 36.7, 36.5, 30.2, 24.9, 18.6, 11.4, 11.3, 1.9. Anal. Calcd for C_15_H_24_AlNO: C, 68.94; H, 9.26; N, 5.36%. Found: C, 69.17; H, 9.44; N, 5.23%.

R^1^ = R^2^ = H, R^3^ = *i*-Bu **C3**. Using a similar procedure to that described for **C1**, but with *i*-Bu_3_Al (1.0 M solution in toluene) as the alkyl aluminum reagent, gave **C3** as a white powder. Yield: 0.33 g, 21%. ^1^H NMR (400 MHz, CDCl_3_, ppm): δ 8.23 (d, *J* = 8.0 Hz, 1H, Py–*H*), 7.67 (d, *J* = 4.0 Hz, 1H, Py–*H*), 7.38–7.34 (m, 1H, Py–*H*), 4.61 (s, 1H, C*H*–O), 2.94–2.80 (m, 2H, C*H*_2_), 1.86–1.67 (m, 3H, C*H*_2_ and Al–CH_2_C*H*(CH_3_)_2_), 1.23 (s, 3H, C*H*_3_), 0.90–0.75 (m, 12H, Al–CH_2_CH(C*H*_3_)_2_), 0.73 (s, 3H, C*H*_3_), −0.07–−0.09 (m, 4H, Al–C*H*_2_CH(CH_3_)_2_). ^13^C NMR (100 MHz, CDCl_3_): δ 164.0, 142.2, 139.6, 133.6, 122.8, 80.4, 35.3, 34.3, 28.2, 28.1, 28.0, 27.9, 26.2, 26.0, 24.5, 22.6, 17.4. Anal. Calcd for C_19_H_32_AlNO: C, 71.89; H, 10.16; N, 4.41%. Found: C, 71.56; H, 10.52; N, 4.67%.

R^1^ = Cl, R^2^ = H, R^3^ = Me **C4**. Using a similar procedure to that described for **C1**, but with **L2** as the pro-ligand, gave **C4** as a white powder. Yield: 1.14 g, 85%. ^1^H NMR (400 MHz, CDCl_3_, ppm): 7.74 (d, *J* = 8.0 Hz, 1H, Py–*H*), 7.44 (d, *J* = 8.0 Hz, 1H, Py–*H*), 4.92 (s, 1H, C*H*–O), 2.85–2.81 (m, 2H, C*H*_2_), 1.92–1.84 (m, 1H, C*H*_2_), 1.71–1.65 (m, 1H, C*H*_2_), 1.51 (s, 3H, C*H*_3_), 0.84 (s, 3H, C*H*_3_), -0.58 (s, 6H, Al–C*H*_3_). ^13^C NMR (100 MHz, CDCl_3_, ppm): *δ* 158.4, 145.5, 142.4, 132.8, 126.7, 81.5, 36.1, 35.8, 31.6, 29.5, 23.9, 18.1, 14.3, −4.5. Anal. Calcd for C_13_H_19_AlClNO: C, 58.32; H, 7.15; N, 5.23%. Found: C, 58.11; H, 7.24; N, 5.38%.

R^1^ = H, R^2^ = R^3^ = Me **C5**. Using a similar procedure to that described for **C1**, but with **L3** as the pro-ligand, gave **C5** as a white powder. Yield: 0.36 g, 29%. ^1^H NMR (400 MHz, Toluene-*d*_8_, ppm): δ 7.49 (d, *J* = 4.0 Hz, 1H, Py–*H*), 6.74 (d, *J* = 8.0 Hz, 1H, Py–*H*), 6.51–6.48 (m, 1H, Py–*H*), 2.21–2.18 (m, 2H, C*H*_2_), 1.56–1.51 (m, 1H, C*H*_2_), 1.49 (s, 3H, C*H*_3_), 1.43 (s, 3H, C*H*_3_), 1.22–1.15 (m, 1H, C*H*_2_), 0.83 (s, 3H, C*H*_3_), −0.10 (s, 3H, Al–(C*H*_3_)_2_), −0.30 (s, 3H, Al–(C*H*_3_)_2_). ^13^C NMR (100 MHz, toluene-*d*_8_, ppm): δ 163.2, 143.4, 142.5, 134.3, 126.0, 87.7, 40.3, 36.72, 28.9, 27.8, 25.2, 23.3, −2.09, −2.92. Anal. Calcd. for C_14_H_22_AlNO: C, 67.99; H, 8.97; N, 5.66%. Found: C, 68.20; H, 8.83; N, 5.79%.

R^1^ = Cl, R^2^ = R^3^ = Me **C6**. Using a similar procedure to that described for **C1**, but with **L4** as the pro-ligand, gave **C6** as a white powder. Yield: 0.48 g, 34%. ^1^H NMR (300 MHz, CDCl_3_, ppm): δ 7.52 (d, *J* = 9.0 Hz, 1H, Py–*H*), 7.28 (t, *J* = 9.0 Hz, 1H, Py–*H*), 2.88–2.83 (m, 2H, C*H*_2_), 2.02–1.91 (m, 1H, C*H*_2_), 1.70–1.62 (m, 1H, C*H*_2_), 1.44 (s, 3H, C*H*_3_), 1.29 (s, 3H, C*H*_3_), 0.91 (s, 3H, C*H*_3_), −0.69 (s, 6H, Al–(C*H*_3_)_2_). ^13^C NMR (100 MHz, CDCl_3_, ppm): *δ* 161.1, 146.7, 141.1, 128.2, 125.0, 81.0, 37.4, 33.8, 27.3, 25.1, 23.2, 21.0, 14.4, −8.3. Anal. Calcd for C_14_H_21_AlClNO: C, 59.68; H, 7.51; N, 4.97%. Found: C, 59.55; H, 7.32; N, 4.85%.

### 2.4. X-ray Crystallographic Studies

Single crystal X-ray diffraction data for **C1** and **C4** were collected on a Rigaku Sealed Tube CCD (Saturn 724+) diffractometer (Tokyo, Japan) with graphite monochromated Mo-Kα radiation (*λ* = 0.71073 Å) at 293(2) K. Cell parameters were obtained by global refinement of the positions of all collected reflections. Intensities were corrected for Lorentz and polarization effects and empirical absorption. The structures were solved by direct methods and refined by full-matrix least squares on *F*^2^. All hydrogen atoms were placed in calculated positions. Structure solution and refinement were performed using the SHELXTL-97 package [38,39]. Details of the X-ray structure determinations and refinements are provided in Table 1. The Cambridge Crystallographic Data Centre CCDC 1851289 and 1851290 for **C1** and **C4,** respectively, contain the supplementary crystallographic data for the paper. These data can be obtained free of charge from the Cambridge Crystallographic Data Centre (Cambridge, UK).

### 2.5. General Procedure for ε-Caprolactone Polymerization

A typical polymerization procedure in the presence of one equivalent of benzyl alcohol is outlined as follows. Precatalyst **C1** (0.0047 g, 0.020 mmol) was dissolved in toluene (2 mL) in a Schlenk flask at room temperature and a solution of benzyl alcohol (0.020 mmol) in toluene added and the mixture stirred at room temperature for 5 min. The flask was then placed in a temperature-controlled oil bath preheated to 90 °C and ε-CL (0.571 g, 5.0 mmol) was injected. After the solution was stirred for the pre-determined time, the polymerization was terminated by the addition of glacial acetic acid (ca. 0.2 mL). The resulting viscous solution was diluted with dichloromethane and then transferred to a beaker containing cold methanol (100 mL) with stirring. The resultant polymer was collected on filter paper and dried under reduced pressure to give a white solid.

## 3. Results and Discussion

### 3.1. Synthesis and Characterization of ***C1***–***C6***

**Scheme 1:**
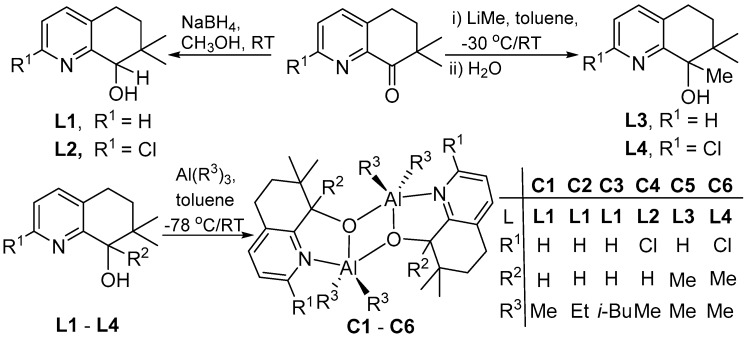
Synthetic routes to **L1**–**L4** and aluminum complexes **C1**–**C6.**

Based on previous synthetic approaches, reduction of the 5,6-dihydro-7,7-dimethylquinolin-8-ones, 2-R^1^-7,7-Me_2_C_9_H_6_N-8-O (R^1^ = H; R^1^ = Cl), with NaBH_4_ or LiMe gave the dihydroquinolin-8-ols, 2-R^1^-7,7-Me_2_,8-R_2_C_9_H_6_N-8-OH (R^1^ = R^2^ = H **L1**; R^1^ = Cl, R^2^ = H **L2**; R^1^ = H, R^2^ = Me **L3**; R^1^ = Cl, R^2^ = Me **L4**), in high yield (Scheme 1). All four compounds have been fully characterized by ^1^H/^13^C NMR, FT-IR spectroscopy as well as by elemental analysis. Treatment of **L1** with one equivalent of (R^3^)_3_Al (R^3^ = Me, Et, *i*-Bu) in toluene at −78 °C afforded [{2-R^1^-7,7-Me_2_-8-R_2_C_9_H_6_N-8-O}AlR^3^_2_]_2_ (R^1^ = R^2^ = H, R^3^ = Me **C1**; R^1^ = R^2^ = H, R^3^ = Et **C2**; R^1^ = R^2^ = H, R^3^ = i-Bu **C3**), while reaction of **L2**, **L3** and **L4** with solely Me_3_Al gave [{2-R^1^-7,7-Me_2_-8-R^2^C_9_H_6_N-8-O}AlMe_2_]_2_ (R^1^ = Cl, R^2^ = H **C4**; R^1^ = H, R^2^ = Me **C5**; R^1^ = Cl, R^2^ = Me **C6**), in moderate to good yields. All six complexes have been characterized by ^1^H and ^13^C NMR spectroscopy as well as by elemental analysis. In addition, **C1** and **C4** have been the subject of single crystal X-ray diffraction studies.

**Figure 1:**
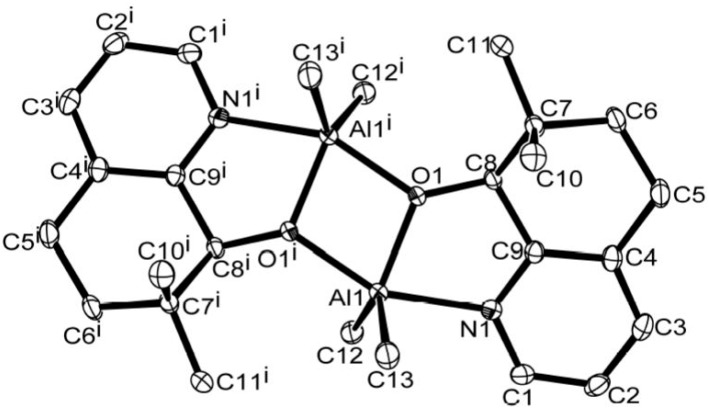
ORTEP representation of **C1**. Thermal ellipsoids are shown at 30% probability while hydrogen atoms have been omitted for clarity.

**Figure 2:**
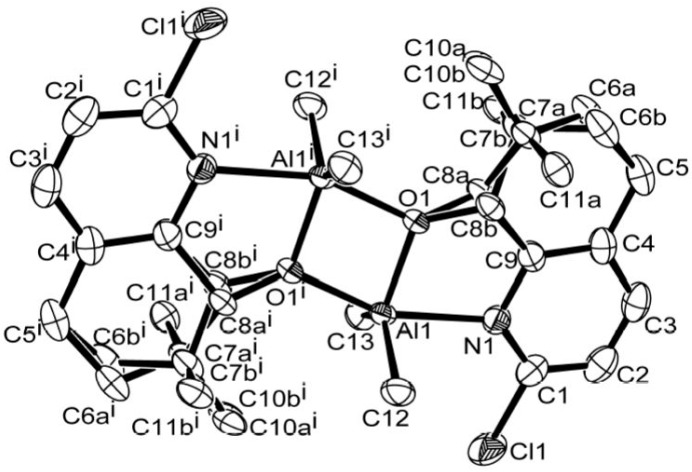
ORTEP representation of **C4** showing the disorder in the saturated sections of the rings. Thermal ellipsoids are shown at 30% probability while hydrogen atoms have been omitted for clarity.

Single crystals of **C1** and **C4** suitable for the X-ray determinations were grown by the slow diffusion of *n*-hexane into their toluene solutions at room temperature under a nitrogen atmosphere. Perspective views of the structures of **C1** and **C4** are shown in Figure 1 and Figure 2; selected bond lengths and bond angles are collected in Table 2. Both **C1** and **C4** have been generated by symmetry and adopt similar arrangements and will be discussed together. Their structures consist of two aluminum centers that are each chelated by an N^O-bound 5,6-dihydro-7,7-dimethylquinolin-8-olate which also acts as a bridging ligand via O1. The five-coordinate geometry of each metal center is completed by two methyl ligands. The main difference between the structures relates to the substituent located on the 2-position of the N^O-chelate *viz.* H (**C1**) and Cl (**C4**). Indeed, this dimeric arrangement is similar to that observed in the unsubstituted 5,6-dihydroquinolin-8-olates **M** (Chart 1) [37]. The Al-N bond length in **C1** [2.122(13) Å] is slightly shorter than that in **C4** [2.194(3) Å] likely reflecting the steric properties of the more bulky chloride substituent positioned at **C1**. There are no intermolecular contacts of note.

The ^1^H NMR spectra of **C1**–**C6** reveal characteristic peaks for the corresponding 5,6-dihydro-7,7-dimethylquinolin-8-olate ligands with the inequivalent *gem*-dimethyl groups giving rise to two distinct signals. Likewise, the Al-CH_3_ (**C1**, **C4**–**C6**) or Al-CH_2_ (**C2**, **C3**) resonances are seen, in most cases, as separate signals and appear most upfield in their spectra. Interestingly, the ^1^H NMR spectrum of **C1**, recorded in three different deuterated solvents, indicates that the solvent polarity has some effect on the chemical shift of the resonances (Table 3). In an apolar solvent such as toluene-*d*_8_ and benzene-*d*_6_, each proton displays very similar chemical shifts. However, the shift in chloroform-*d* is greatly affected, which is best exemplified by the most upfield Cy-CH_3_ signal which appears at δ 0.58 in C_6_D_6_ while in CDCl_3_ at δ 0.82. In addition, the number of Al-CH_3_ signals shows some variation with two peaks (δ −0.12, −0.26) in C_6_D_6_ and three peaks (δ −0.62, −0.66, −0.67) in CDCl_3_. It is uncertain as to the origin of these differences but it may be due to the ability of the more polar chloroform to interrupt the apparent monomeric/dimeric equilibrium in solution (*vide infra*).

### 3.2. Ring Opening Polymerization of ε-Caprolactone by ***C1***–***C6***

To explore the capacity of **C1**–**C6** to serve as pro-initiators for the ring-opening polymerization of ε-CL, **C1** was firstly selected as the test system; the results of the catalytic evaluations are collected in Table 3. In the first instance, the study focused on establishing the optimal temperature by performing the polymerization runs between 25 and 110 °C. Typically, each run was conducted in toluene with one equivalent of PhCH_2_OH (BnOH) as activator and 250 equivalents of the monomer over a run time of 10 min (runs 1–7, Table 4). Below 40 °C, there was no monomer consumption after 10 min. However, on raising the temperature from 60 to 90 °C, the conversion gradually increased from 47% at 60 °C to a maximum of >99% at 90 °C (runs 4, 5, Table 4). Further increasing the temperature up to 110 °C led to a steady decrease in the conversion. These findings contrast with observations seen for **M** (Chart 1) in which only 20% conversion was observed at 80 °C and 95% at 90 °C [37].

With the ε-CL:Al:BnOH ratio fixed at 250:1:1 and the run temperature at 90 °C, the influence of reaction time on the polymerization was explored (runs 5, 8–10, Table 4). Monitoring the polymerization by ^1^H NMR spectroscopy at intervals of 3, 5, 7 and 10 min saw the conversion rise from 43% to approaching 100% while the molecular weight of polymer increased from 1.04 × 10^4^ to 1.89 × 10^4^ g/mol. By contrast, the TOF decreased with time implying gradual deactivation of the active species over longer run times. To investigate the importance of BnOH in the activation of **C1**, the polymerization runs were performed with and without benzyl alcohol over different run times but with the temperature maintained at 90 °C; the results are collected in Table 4 (runs 11–17). Without benzyl alcohol, **C1** showed no activity after 20 min while after 30 min 15% conversion was observed, highlighting the induction period needed when using **C1** alone. This result contrasts with the virtually 100% conversion observed after 10 min using an equimolar ratio of **C1** to BnOH (run 5, Table 4). On increasing the BnOH/Al ratio from 1 to 10, the conversion gradually decreased from 99 to 69% (runs 5, 14–17, Table 4) while the *M*_n_ of the PCL lowered from 1.89 × 10^4^ to 0.28 × 10^4^ g/mol, suggesting the possible role of BnOH as a chain transfer reagent. An alternative explanation could be due to the likely intermediate ‘**L1**Al(OCH_2_Ph)_2_’ undergoing disproportionation and dimerization to form an inactive species in a manner similar to that previously reported [41].

**Figure 3:**
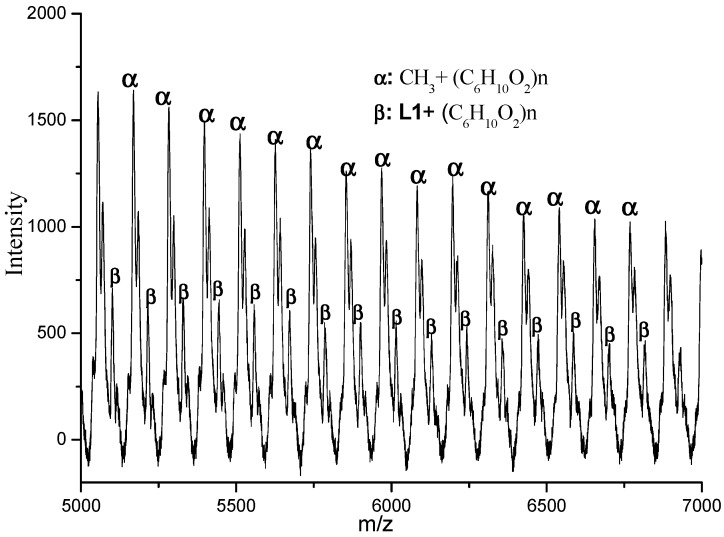
The MALDI-TOF mass spectrum of the PCL obtained using **C1** (run 13, Table 3).

**Figure 4:**
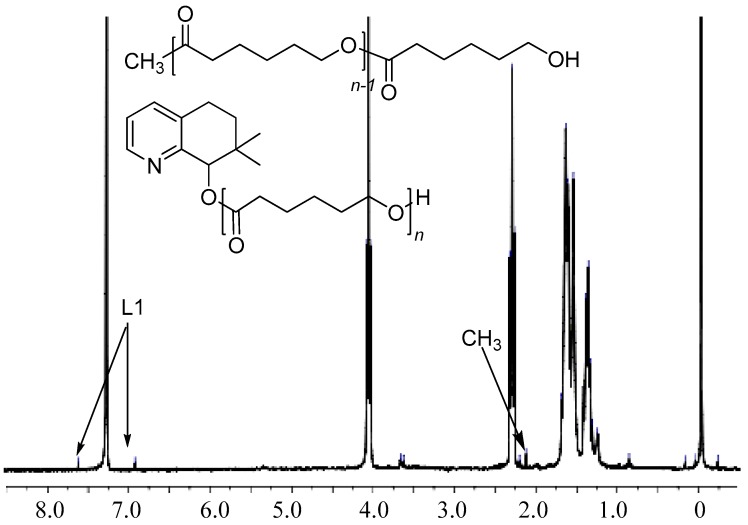
The ^1^H NMR spectrum of the PCL obtained using **C1** as initiator (run 13, Table 3).

**Scheme 2:**
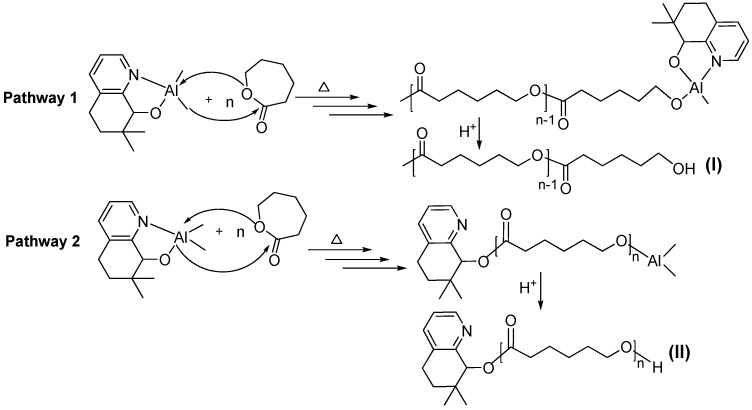
Possible mechanistic pathways for the ring opening polymerization of ε-CL using **C1**.

In order to gain some information on the structural features of the PCLs, all the samples obtained by employing different amounts of BnOH were analyzed by ^1^H NMR spectroscopy and by MALDI-TOF mass spectrometry. In the case where no BnOH was employed, the MALDI-TOF spectrum indicated that the polymer was comprised of linear polymers capped by two different types of end-group (**α** and **β** in Figure 3). In **α** (the major species) the capping group is CH_3_ while in **β** it is **L1**. These findings are supported by the ^1^H NMR data that reveal aromatic signals characteristic of **L1** in **β** and a distinct singlet peak at δ 2.14 that can be assigned to the acetyl end group (CH_3_C=O) in **α** (Figure 4). Therefore, it would seem probable that this polymerization proceeded by a coordination-insertion mechanism in which the Al-Me and Al-**L1** independently initiated the ring opening polymerization by pathways **I** and **II** (Scheme 2) [42,43,44,45].

**Figure 5:**
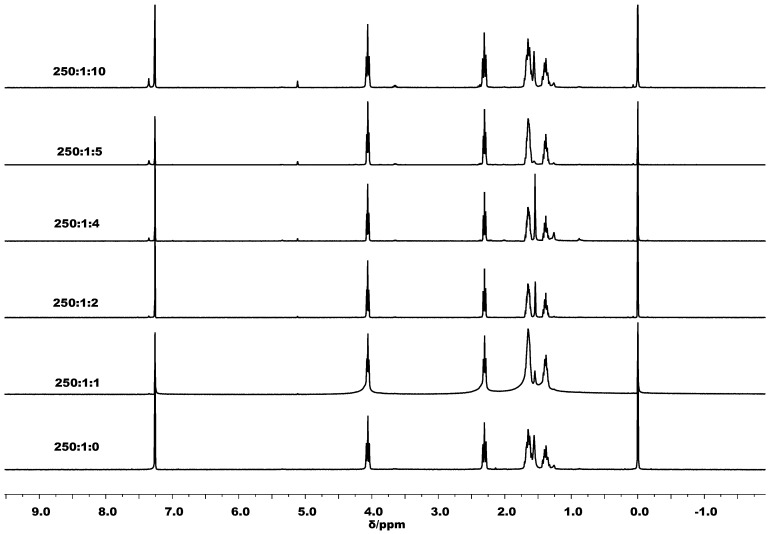
The ^1^H NMR spectra of the PCL obtained using **C1** with different amounts of BnOH (runs 5, 13–17, Table 4).

**Figure 6:**
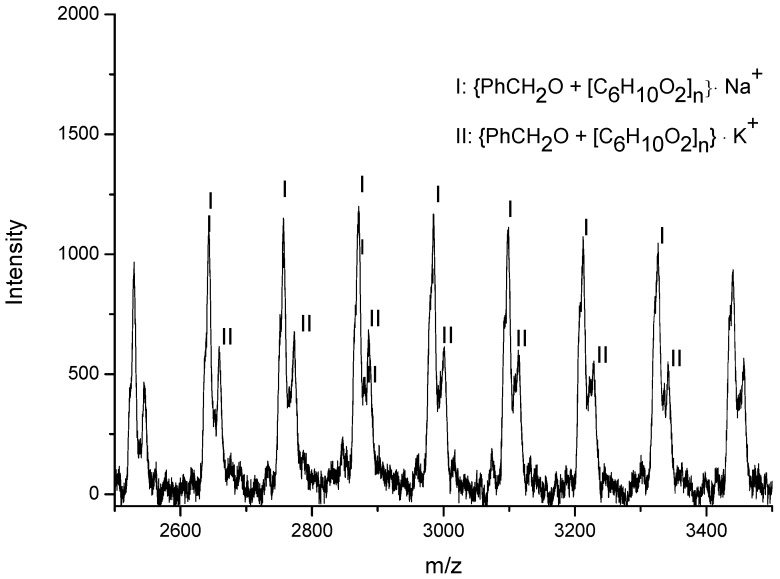
The MALDI-TOF mass spectrum of the PCL obtained using **C1**/BnOH as initiator (run 5, Table 4).

On the other hand, when one equivalent or more of BnOH was employed in combination with **C1**, the ^1^H NMR spectra of the PCLs showed only signals typical of BnO end groups (e.g., δ 5.0 (OC*H*_2_Ph) in Figure 5). This observation was corroborated by the MALDI-TOF spectrum that showed solely peaks corresponding to a linear chain polymer capped by a BnO group (Figure 6). Similarly, the polymers obtained by using larger amounts of BnOH possessed linear structures with BnO end groups, which were again confirmed by ^1^H NMR spectroscopy and MALDI-TOF mass spectrometry (Figures S1–S4). As an additional feature of the spectra, it was evident that the more BnOH employed the lower the molecular weight of the polymer formed (runs 5, 14–17, Table 4).

With the amount of benzyl alcohol maintained at one molar equivalent, the effect of changing the amount of ε-CL on the performance of **C1** was then examined. On increasing the molar ratio of ε-CL:Al from 100 to 300, all the conversions were greater than 90% with the TOF showing an upward trend (runs 5, 18, 19, Table 4). However, on further increasing the ε-CL:Al ratio between 400 and 750, a dramatic decrease of both the conversion and TOF was evident (runs 20–22, Table 4). On the other hand, the molecular weight of the PCL was found to gradually increase from 0.75 × 10^4^ to 2.51 × 10^4^ g/mol as the ε-CL:Al molar ratio was raised from 100 to 750 (runs 5, 18–22, Table 4), which can be explained by the faster rate of coordination and rate of propagation at higher monomer concentration.

The effect of solvent on the polymerizations using **C1** was also studied. As a bulk polymerization was conducted at 90 °C, almost 100% conversion was observed after 10 min. The molecular weight was, however, much lower than that obtained in toluene (run 23, Table 4). When using n-hexane, dichloromethane or THF, the activity dramatically decreased and only trace amounts of polymer could be obtained (runs 24–26, Table 4). It is worthy to note that the above findings are significantly different to that found using analogue **M** (Chart 1). In that case, no activity was observed in bulk polymerization and moderate conversion was observed in other solvents [37]. One reason to account for these differences may be due to the improved solubility of **C1** leading to more facile reactivity.

Based on the optimal conditions established for **C1**, the remaining five complexes, **C2**–**C6**, were also investigated for ROP; the results are collected in Table 4 alongside the data for **C1**. In terms of the steric properties of the aluminum-R^3^ group, replacing Me with an *i*-Bu group slightly decreased the conversion from 99 to 91% and the molecular weight from 1.89 × 10^4^ to 1.21 × 10^4^ g/mol (runs 1–3, Table 5); the increased reactivity of an Al-Me over an Al-*i*-Bu towards BnOH may be a contributing factor. With a chloride present at the 2-position of the ligand, the conversion decreased rapidly as exemplified by **C1** > **C4** and **C5** > **C6**. The reason for this observation is likely due to the electron withdrawing properties of a chloride resulting in decreased electron density at aluminum and in-turn slower reaction; steric factors could no doubt also be influential. In addition, the molecular weight of the PCLs generated by **C5** and **C6** (R^2^ = Me) are slightly lower than that seen with **C1**–**C4** (R^2^ = H), suggesting the detrimental effect of the Cy-C*MeO* methyl group on chain propagation. Similar to **C1**, the MALDI-TOF mass spectra of the PCL generated by **C2**–**C5** showed linear polymers capped with BnO groups as the unique polymer class. Likewise, the ^1^H NMR spectra of PCLs generated by **C2**–**C5** also clearly showed signals characteristic of a BnO group (Figures S5–S9). Indeed, these results are consistent with the polymerizations proceeding by ‘coordination-insertion’ route with ‘LAl-OCH_2_Ph’ presumed as the active species. Unexpectedly, the structural analysis of the PCL obtained by **C6**/BnOH indicated that two polymer families were present, the major one being a linear structure capped with a PhCH_2_O group and the other a linear one capped with a methoxy group. These results suggest that the different R^1^ and R^2^ substituents have little effect on the polymer structure but have large effect on their reactivity.

### 3.3. Mechanistic Analysis

On account of the differences in polymerization behavior between **C1**–**C6** and its unsubstituted counterpart **M** (Chart 1), the mode of activation was subject to a new ^1^H and ^27^Al NMR spectroscopic investigation. In particular, toluene-*d*_8_ solutions of **C1**, **C1**/BnOH and **C1**/BnOH in the presence of 10 molar equivalents of ε-CL, were examined at various temperatures.

**Figure 7:**
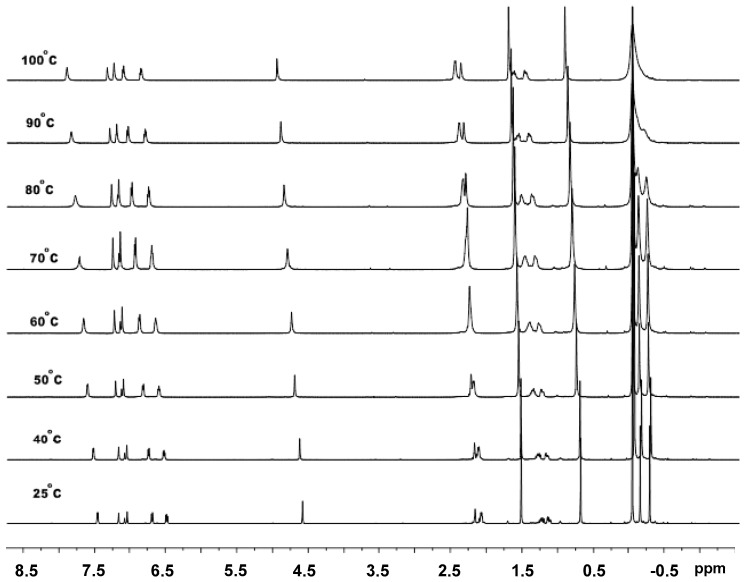
VT-^1^H NMR spectra of **C1** recorded between 25 and 100 °C (in toluene-*d*_8_).

**Figure 8:**
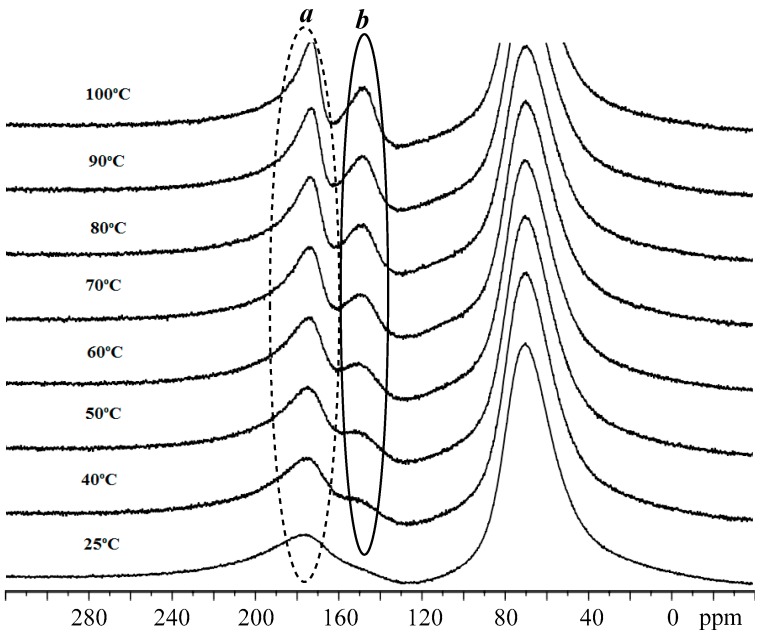
VT-^27^Al NMR spectra of **C1** recorded between 25 and 100 °C (in toluene-*d*_8_); the broad signal at δ 68.0 derives from the aluminum alloy present in the NMR probe.

Firstly, the variable temperature ^1^H NMR experiment was performed on **C1** alone; the stacked spectra are shown in Figure 7. The data reveal the signals for the ligand show some modest shift to lower field with increasing temperature. On the other hand, two separate singlet peaks (δ −0.11, −0.24) for the Al-Me’s at room temperature significantly shifted to lower field and became progressively broader on increasing the temperature. When the temperature reached 100 °C, two peaks effectively collapsed at ca. δ 0, which may be due to the onset of an exchange process at high temperature [46]. In the ^27^Al NMR spectrum of **C1**, one broad peak around δ 176.83 (***a***) was evident at room temperature. However, on raising the temperature from 25 to 100 °C, a second more upfield peak (***b***) (δ 127.9) gradually grew in intensity (Figure 8). Based on these findings, we presume that **C1** exists mainly as a dimeric species at low temperature which is partly transformed into a monomeric species at higher temperature in a manner similar to that previously reported [37].

**Figure 9:**
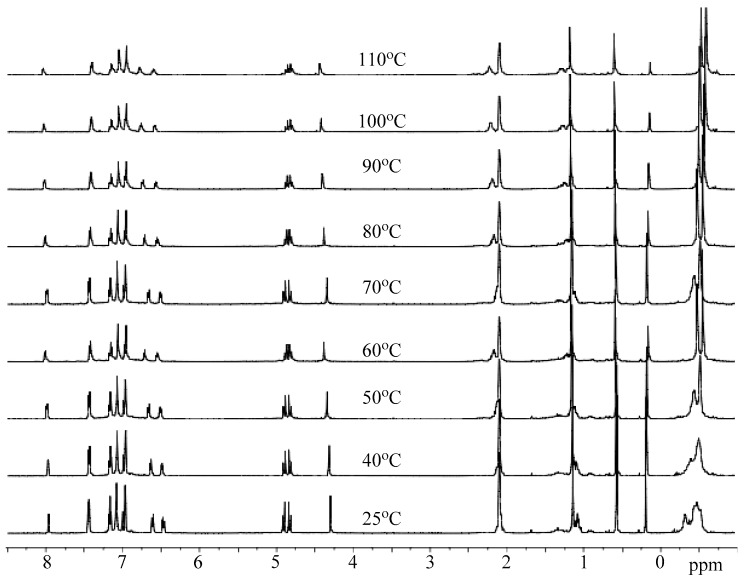
VT-^1^H NMR spectra of **C1**/BnOH recorded between 25 and 110 °C (in toluene-*d*_8_).

**Figure 10:**
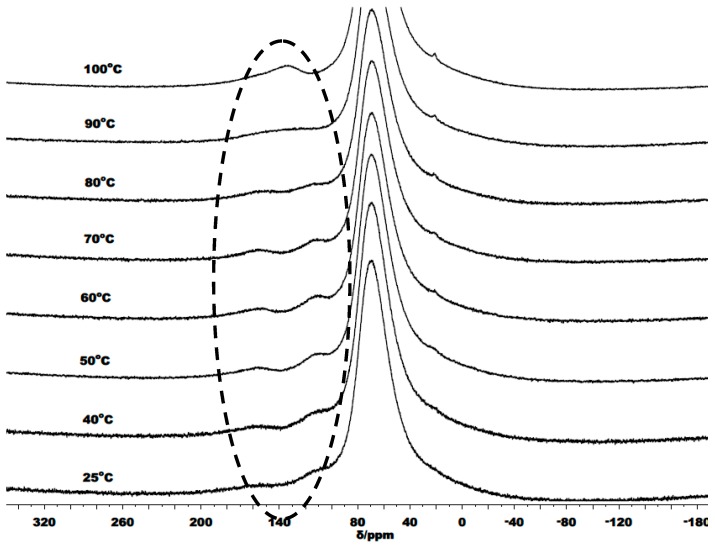
VT-^27^Al NMR spectra of **C1**/BnOH recorded between 25 and 100 °C (in toluene-*d*_8_); the broad signal at δ 68.0 derives from the aluminum alloy present in the NMR probe.

We also monitored the variable temperature ^1^H and ^27^Al NMR spectra of a 1:1 mixture of **C1** and BnOH; the corresponding spectra recorded between 25 and 100 °C are collected in Figure 9 and Figure 10. The ^1^H NMR spectrum of **C1**/BnOH shows multiple upfield peaks for the Al-Me resonances at room temperature which converged into two sharp peaks on raising the temperature. By contrast, there were no major shifts of the downfield H_aryl_ protons over the temperature range. At room temperature, the coordinated benzyl alcohol methylene protons are inequivalent leading two mutually coupled doublets, this splitting pattern is maintained up to 70 °C above which the signals start to merge and broaden. Unexpectedly, the ratio of the PhC*H*_2_O:Al-C*H*_3_ protons is close to 2:6 at each temperature, which would appear to rule out a species of the type **L1**Al(OCH_2_Ph)(CH_3_). In the ^27^Al NMR spectra of **C1**/BnOH, two broad peaks were observed in the temperature range 25 to 80 °C. Further increasing the temperature to 90 °C led to apparent coalescence and then at 100 °C the formation of a single broad peak. By analogy with the NMR findings for **C1** alone, we propose an equilibrating mixture of dimeric and monomeric species each coordinated by intact BnOH ligands that becomes time-averaged at high temperature.

**Figure 11:**
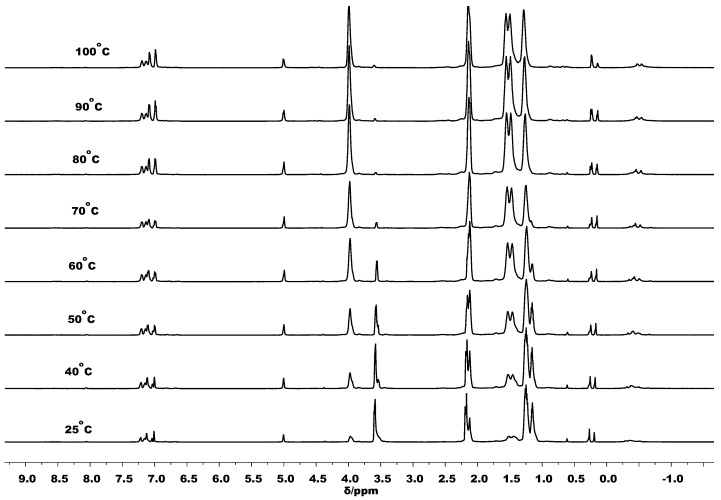
VT-^1^H NMR spectra of **C1/**BnOH/10 ε-CL recorded at temperatures between 25 and 100 °C (in toluene-*d*_8_).

In order to gain information about the catalyst when in the presence of monomer, the ^1^H and ^27^Al NMR spectra of **C1**/BnOH in the presence of 10 equivalents of ε-CL were also conducted over the 25 to 100 °C temperature range; the spectra are shown in Figure 11. In all the VT ^1^H NMR spectra the PhC*H*_2_O resonance appeared as a singlet (around δ 5.0), which is indicative of a PhCH_2_O end group in PCL. Hence, it is apparent that the polymerization occurred rapidly when the monomer was mixed with **C1**/BnOH even at room temperature. As the temperature was raised, the peaks for PCL (δ 3.94, 1.52) gradually increased as the resonance for ε-CL (δ 3.58) reduced, in accord with the higher polymerization rate at higher temperature. On the other hand, the VT-^27^Al NMR spectra /BnOH/10 ε-CL gave a single broad resonance at δ 69.0 across the temperature range that we tentatively assign to the active species; unfortunately, this chemical shift also coincides with the probe signal. Nevertheless, related aluminum complexes containing bound α-alkoxy esters, that have been considered as the active species in the ROP, have also shown resonances around δ 70 in their ^27^Al NMR spectra [13].

## 4. Conclusions

A series of soluble aluminum 5,6-dihydro-7,7-dimethylquinolin-8-olates, **C1**–**C6**, have been successfully prepared and fully characterized. The molecular structures of **C1** and **C4** indicate that they adopt dimeric forms in the solid state. In the absence of BnOH, **C1** showed only low catalytic efficiency for the ROP of ε-CL with the spectroscopic and spectrometric data for the resulting polymer in agreement with a linear structure capped with either a methyl or a **L1** group. By contrast, in the presence of BnOH, **C1** exhibited excellent efficiency for the ROP of ε-CL with essentially 100% conversion in only 10 min at 90 °C. The chloro-substituted complexes **C4** and **C6** showed lower activity than that seen with **C1**–**C3** and **C5**, highlighting the importance of both electronic and steric factors on initiator performance. All polymers displayed linear structures that were capped with PhCH_2_O groups, which was confirmed by ^1^H NMR spectroscopy and by MALDI-TOF mass spectrometry. The solution state properties of **C1** and **C1**/BnOH over various temperatures have been investigated by multinuclear NMR spectroscopy and highlight the importance of both monomeric and dimeric species. In the presence of 10 molar equivalents of monomer, the active initiator generated from **C1**/BnOH, could be monitored by ^27^Al NMR spectroscopy while the chain propagation by ^1^H NMR spectroscopy.

## Figures and Tables

**Table 1:** Crystal data and structure refinement for **C1** and **C4.**

	C1	C4
Empirical formula	C_26_H_40_Al_2_N_2_O_2_	C_13_H_19_AlNOCl
Formula weight	466.56	267.72
Crystal color	colorless	colorless
Temperature (K)	293(2)	293(2)
Wavelength (Å)	0.71073	0.71073
Crystal system	monoclinic	monoclinic
space group	P2/n	P21/n
a (Å)	9.762(2)	10.934(5)
b (Å)	9.773(2)	9.902(4)
c (Å)	13.676(3)	13.019(6)
α (°)	90.00	90.00
β (°)	90.39(3)	100.391(5)
γ (°)	90.00	90.00
Volume (Å^3^)	1304.7(5)	1386.5(11)
Z	2	4
D_calc_ (Mg m^−3^)	1.188	1.283
µ (mm^−1^)	0.136	0.323
F (000)	504.0	568.0
Crystal size (mm)	0.49 × 0.29 × 0.21	0.64 × 0.38 × 0.16
θ range (°)	5.14–54.96	4.49–54.94
Limiting indices	−12 ≤ h ≤ 11 −12 ≤ k ≤ 12 −17 ≤ l ≤ 17	−14 ≤ h ≤ 14 −12 ≤ k ≤ 12 −16 ≤ l ≤ 16
No.of rflns collected	13159	14473
No. of unique rflns R_int_	2963 (0.0301)	3144 (0.0526)
Completeness to θ (%)	99.0%	99.3%
Goodness-of-fit on F^2^	1.198	1.137
Final R indices [I > 2σ (I)]	R_1_ = 0.0372, wR_2_ = 0.1078	R_1_ = 0.0652, wR_2_ = 0.1592
R indices (all data)	R_1_ = 0.0429, wR_2_ = 0.1246	R_1_ = 0.0678, wR_2_ = 0.1621
Largest diff. peak and hole (eÅ^−3^)	0.44/−0.48	0.36/−0.34

**Table 2:** Selected bond lengths (Å) and angles (°) for **C1** and **C4.**

	C1	C4
Bond lengths (Å)		
Al(1)–N(1)	2.122(13)	2.194(3)
Al(1)–O(1)	1.876(11)	1.856(18)
Al(1)–O(1i)	1.953(11)	1.966(2)
Al(1)–C(12)	1.997(15)	1.977(3)
Al(1)–C(13)	1.983(16)	1.985(3)
O(1)–C(8)	1.427(15)	1.398(5)
Bond angles (°)		
N(1)–Al(1)–O(1)	79.14(5)	78.73(9)
N(1)–Al(1)–O(1i)	154.49(5)	158.62(8)
Al(1)–O(1)–C(8)	121.91(8)	121.8(3)
Al(1)–N(1)–C(9)	113.13(9)	110.97(18)
O(1)–Al(1)–O(1i)	77.25(5)	79.93(8)
C(12)–Al(1)–C(13)	123.79(7)	134.10(13)

The atoms labeled with ‘i’ have been generated by symmetry.

**Table 3:** The ^1^H NMR chemical shifts for **C1** recorded in different deuterated solvents.

Proton Type	δ (ppm) in Toluene-*d*_8_	δ (ppm) in Benzene*-d*_6_	δ (ppm) in Chloroform-*d*
Py-CH	7.44 (1H), 6.63 (1H),6.43 (1H)	7.29 (1H), 6.43 (1H),6.23 (1H)	8.24 (1H), 7.79 (1H), 7.50 (1H)
Cy-CH	4.58 (1H)	4.49 (1H)	4.92 (1H)
Cy-CH_2_	2.04 (2H), 1.20–1.11 (2H)	1.86 (2H), 1.08 (1H),0.96 (1H)	2.88 (2H), 1.94–1.84 (1H),1.74–1.66 (1H)
Cy-CH_3_	1.51 (3H), 0.67 (3H)	1.45 (3H), 0.58 (3H)	1.50 (3H), 0.82 (3H)
Al-CH_3_	−0.12 (3H), −0.26 (3H)	−0.12 (3H), −0.25 (3H)	−0.62, −0.66, −0.67

**Table 4:** The ROP of ε-CL using **C1** ^a^.

Run	ɛ-CL:Al:BnOH	T (°C)	t (min)	Solvent	Conv. (%) ^b^	*M*_n_ (×10^4^) ^c^	*M*_n_ (calc) (×10^4^) ^d^	PDI ^c^	TOF (h^−1^)
1	250:1:1	25	10	Toluene	0				
2	250:1:1	40	10	Toluene	0				
3	250:1:1	60	10	Toluene	47	1.11	1.35	1.27	705
4	250:1:1	80	10	Toluene	85	1.61	2.43	1.47	1275
5	250:1:1	90	10	Toluene	>99	1.89	2.83	1.61	1485
6	250:1:1	100	10	Toluene	95	1.84	2.72	1.72	1425
7	250:1:1	110	10	Toluene	92	1.84	2.63	1.76	1380
8	250:1:1	90	3	Toluene	43	1.04	1.24	1.21	2150
9	250:1:1	90	5	Toluene	63	1.35	1.81	1.41	1890
10	250:1:1	90	7	Toluene	86	1.18	2.46	1.43	1843
11	250:1:0	90	10	Toluene	0				
12	250:1:0	90	20	Toluene	0				
13	250:1:0	90	30	Toluene	15	0.27	0.43	1.15	75
14	250:1:2	90	10	Toluene	96	1.02	2.75	1.47	1440
15	250:1:4	90	10	Toluene	85	0.61	2.43	1.37	1275
16	250:1:5	90	10	Toluene	82	0.51	2.35	1.47	1230
17	250:1:10	90	10	Toluene	69	0.28	1.98	1.15	1035
18	100:1:1	90	10	Toluene	>99	0.75	2.83	1.40	594
19	300:1:1	90	10	Toluene	93	1.58	2.66	1.47	1674
20	400:1:1	90	10	Toluene	63	2.11	1.81	1.57	1512
21	500:1:1	90	10	Toluene	51	2.23	1.46	1.49	1530
22	750:1:1	90	10	Toluene	23	2.51	0.67	1.35	1035
23	250:1:1	90	10	Bulk	>99	0.81	2.83	1.64	1485
24	250:1:1	-	10	Hexane	Trace				
25	250:1:1	-	10	THF	Trace				
26	250:1:1	-	10	CH_2_Cl_2_	9	0.40	0.27	2.03	135
27 ^e^	250:1:1	90	30	Bulk	trace				
28 ^e^	250:1:1	60	30	Hexane	83	1.85	1.90		
29 ^e^	250:1:1	60	30	THF	42	0.80	1.20		
30 ^e^	250:1:1	35	30	CH_2_Cl_2_	29	0.59	1.16		

^a^ Conditions: 20 μmol [Al]; 2 mL solution. ^b^ Determined by ^1^H NMR spectroscopy. ^c^ GPC data were recorded in THF vs. polystyrene standards, using a correcting factor of 0.56 [40]. ^d^ *M*_n_ (calcd) = (monomer/initiator) × (conversion) × 114 + 108 (molar mass of BnOH). ^e^ under reflux.

**Table 5:** The ROP of ε-CL promoted by **C1**–**C6**/BnOH ^a^.

Run	Pro-Initiator (R^1^, R^2^, R^3^)	Conv. (%) ^b^	*M*_n_ (×10^4^) ^c^	*M*_n_ (calc.) (×10^4^) ^d^	PDI ^c^	TOF (h^−1^)
1	**C1** (H, H, Me)	>99	1.89	2.83	1.61	1485
2	**C2** (H, H, Et)	95	1.35	2.72	1.32	1425
3	**C3** (H, H, i-Bu)	91	1.21	2.60	1.25	1365
4	**C4** (Cl, H, Me)	83	1.54	2.38	1.55	1245
5	**C5** (H, Me, Me)	97	0.73	2.78	1.30	1455
6	**C6** (Cl, Me, Me)	82	1.24	2.35	1.48	1230

^a^ Conditions: 20 μmol [Al], [ε-CL]:[Al]:[BnOH] = 250:1:1, 2 mL toluene solution, 90 °C, 10 min. ^b^ Determined by ^1^H NMR spectroscopy. ^c^ GPC data were recorded in THF vs. polystyrene standards, using a correcting factor of 0.56 [40]. ^d^ *M*_n_ (calcd) = (monomer/initiator) × (conversion) × 114 + 108 (molar mass of BnOH).

## Supplementary Materials

The following are available online at http://www.mdpi.com/2073-4360/10/7/764/s1, Figures S1–S4: The ^1^H NMR and MALDI-TOF spectrum of PCL obtained by **C1** with different amount BnOH, Figures S5–S9: The MALDI-TOF spectrum of PCL obtained by **C2**–**C6**/BnOH.
