# Supplementary material for: Bimetallic Aluminum 5,6-Dihydro-7,7-dimethyl quinolin-8-olates as Pro-Initiators for the ROP of ε-CL; Probing the Nuclearity of the Active Initiator
Source: Polymers (Basel). 2018 Jul 12;10(7):764. doi: 10.3390/polym10070764 (PMC6403881; doi:10.3390/polym10070764)
Supplement: Supplementary file 1 [file polymers-10-00764-s001.pdf]

Supporting information

Bimetallic aluminum 5,6-dihydro-7,7-dimethylquinolin-8-olates as pro-initiators for the ROP of ε-CL; probing the nuclearity of the active species

Qiurui Zhang, Wenjuan Zhang, Gregory A. Solan, TonglingLiang, and Wen-Hua Sun

Contents

Figure S1-S4: The ^{1}H NMR and MALDI-TOF spectrum of PCL obtained by C1 with different amount BnOH

Figure S1. The ^{1}H NMR and MALDI-TOF spectrum of PCL by **C1+2BnOH**

Figure S2. The ^{1}H NMR and MALDI-TOF spectrum of PCL by **C1+4BnOH**

Figure S3. The ^{1}H NMR and MALDI-TOF spectrum of PCL by **C1+5BnOH**

Figure S4. The ^{1}H NMR and MALDI-TOF spectrum of PCL by **C1+10BnOH**

Figure S5-S9: The MALDI-TOF spectrum of PCL obtained by C2-C6/BnOH

Figure S5. The ^{1}H NMR and MALDI-TOF spectrum of PCL by **C2/BnOH**

Figure S6. The ^{1}H NMR and MALDI-TOF spectrum of PCL by **C3/BnOH**

Figure S7. The ^{1}H NMR and MALDI-TOF spectrum of PCL by **C4/BnOH**

Figure S8. The ^{1}H NMR and MALDI-TOF spectrum of PCL by **C5/BnOH**

Figure S9. The ^{1}H NMR and MALDI-TOF spectrum of PCL by **C6/BnOH**

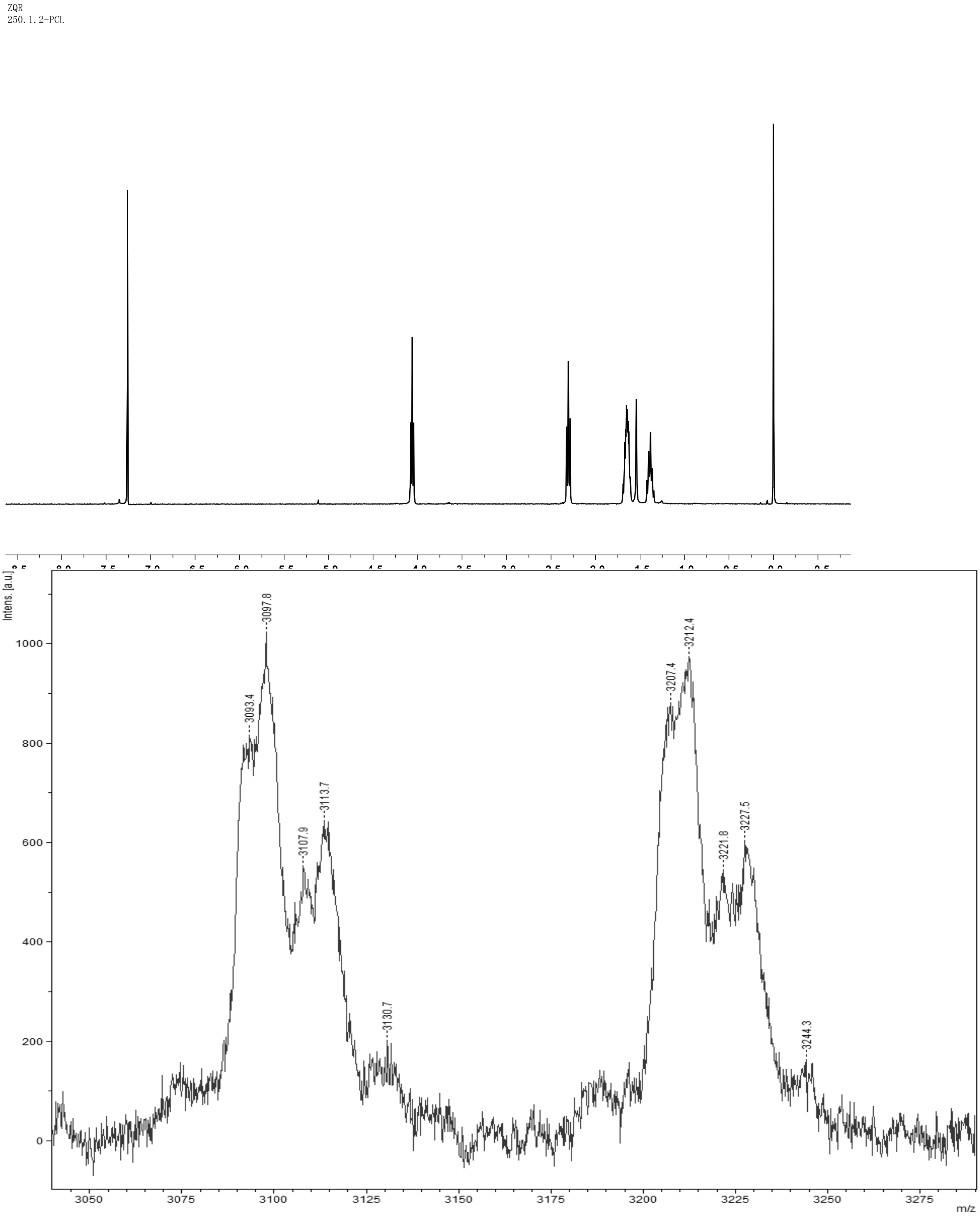

Figure S1 The ^{1}H NMR and MALDI-TOF spectrum of PCL by **C1**+2BnOH.

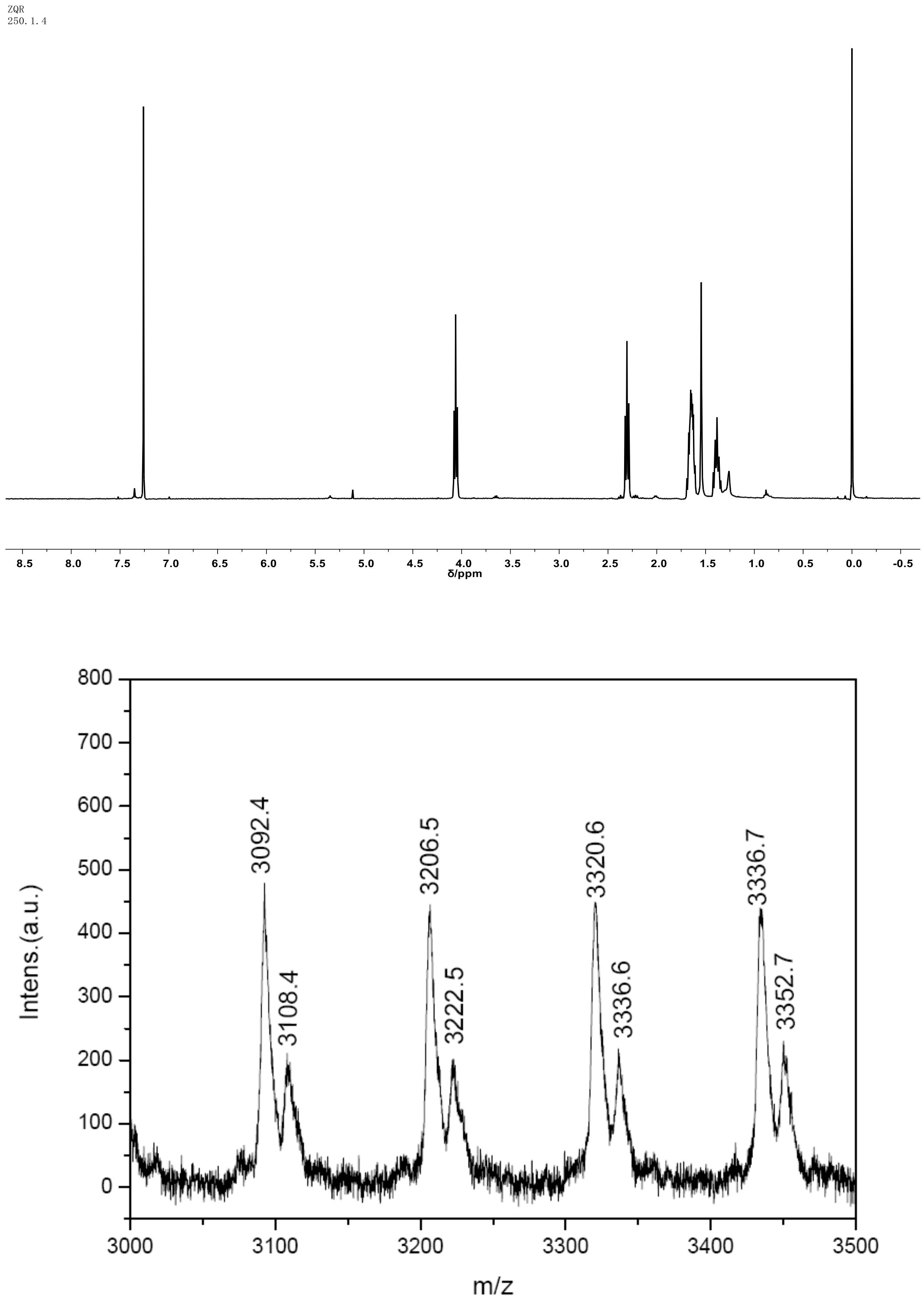

Figure S2 The ^{1}H NMR and MALDI-TOF spectrum of PCL by **C1+4BnOH.**

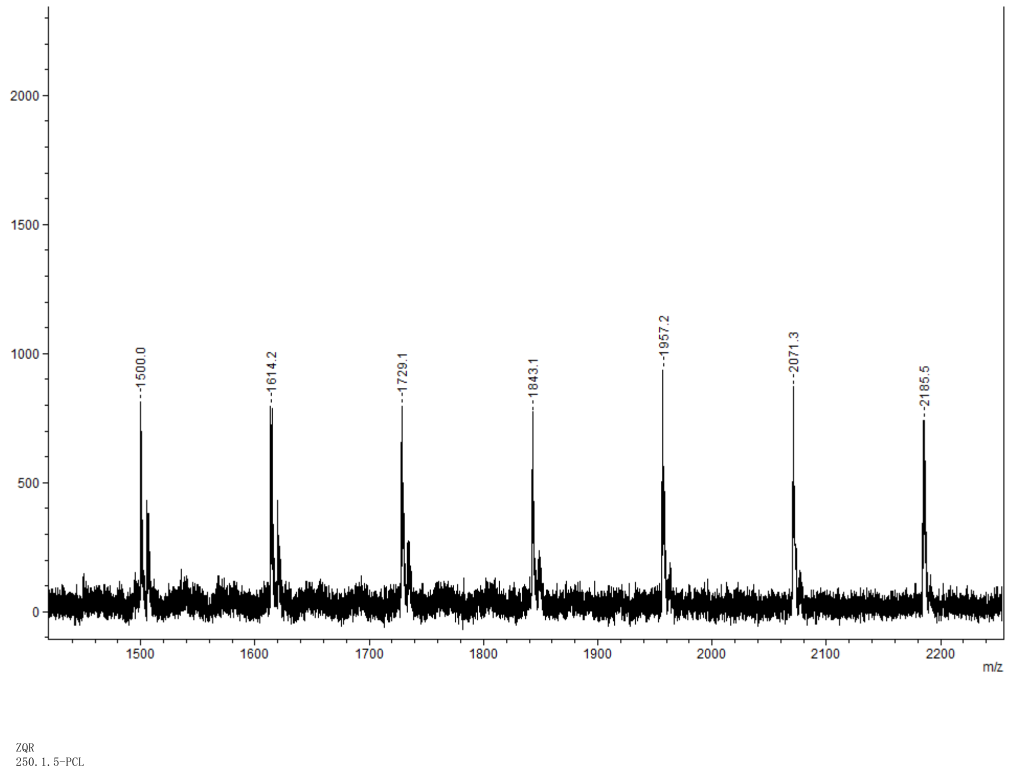

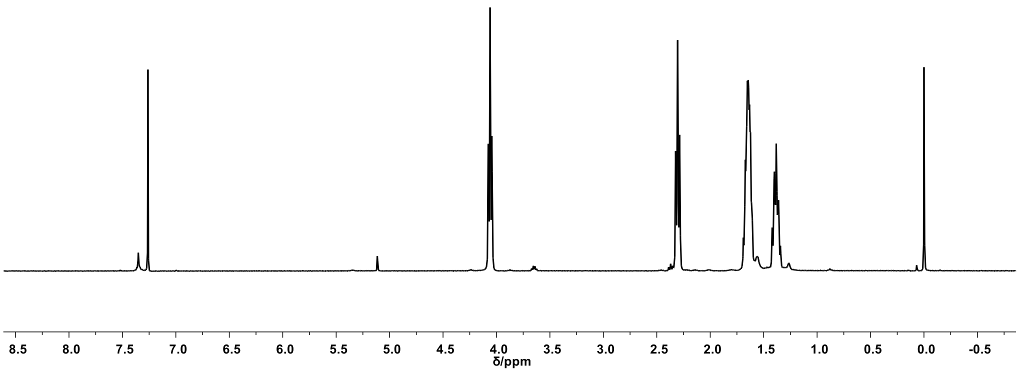

Figure S3 The ^{1}H NMR and MALDI-TOF spectrum of PCL by **C1+5BnOH.**

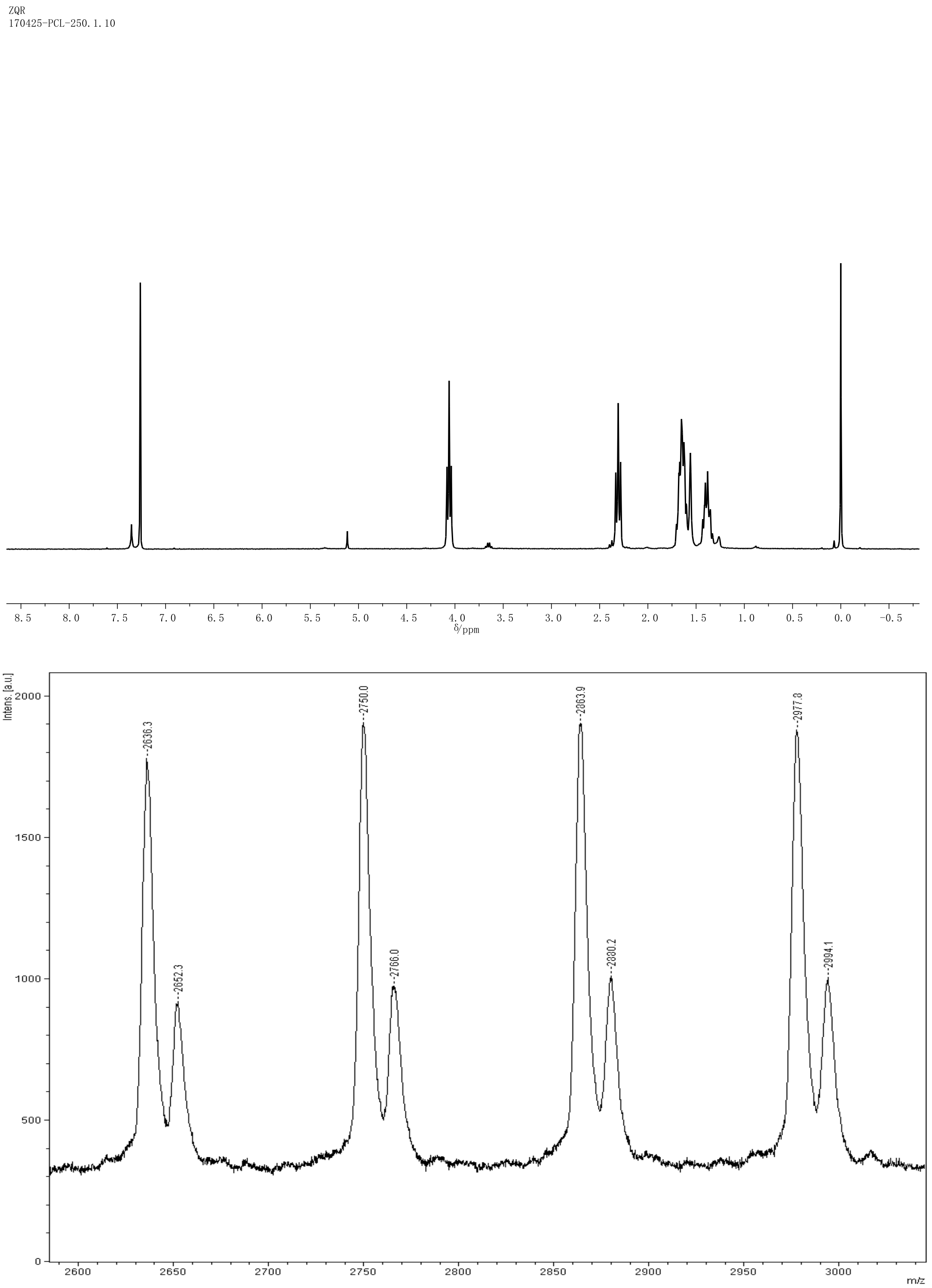

Figure S4 The ^{1}H NMR and MALDI-TOF spectrum of PCL by **C1+10BnOH.**

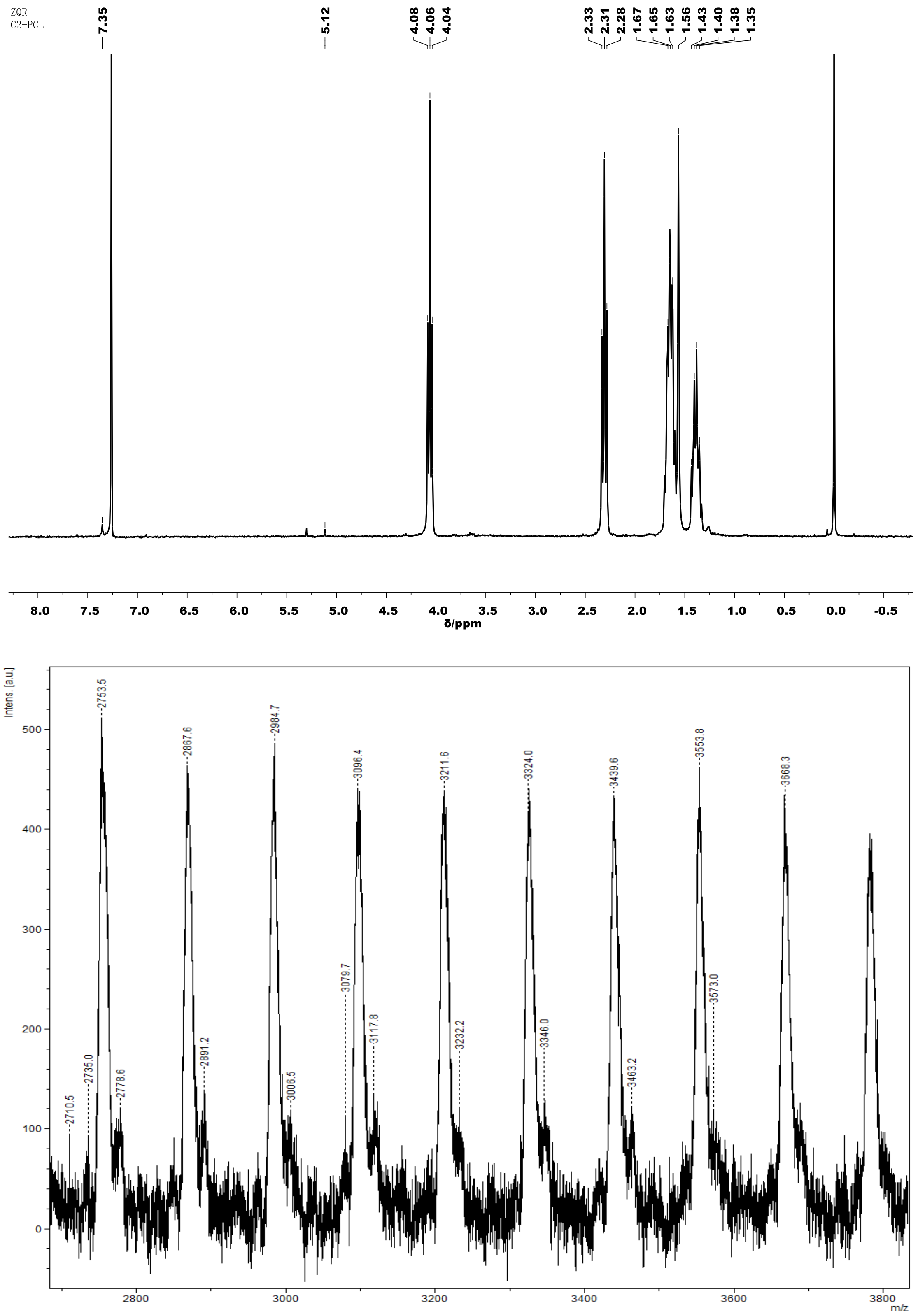

Figure S5 The ¹H NMR and MALDI-TOF spectrum of PCL by **C2/BnOH.**

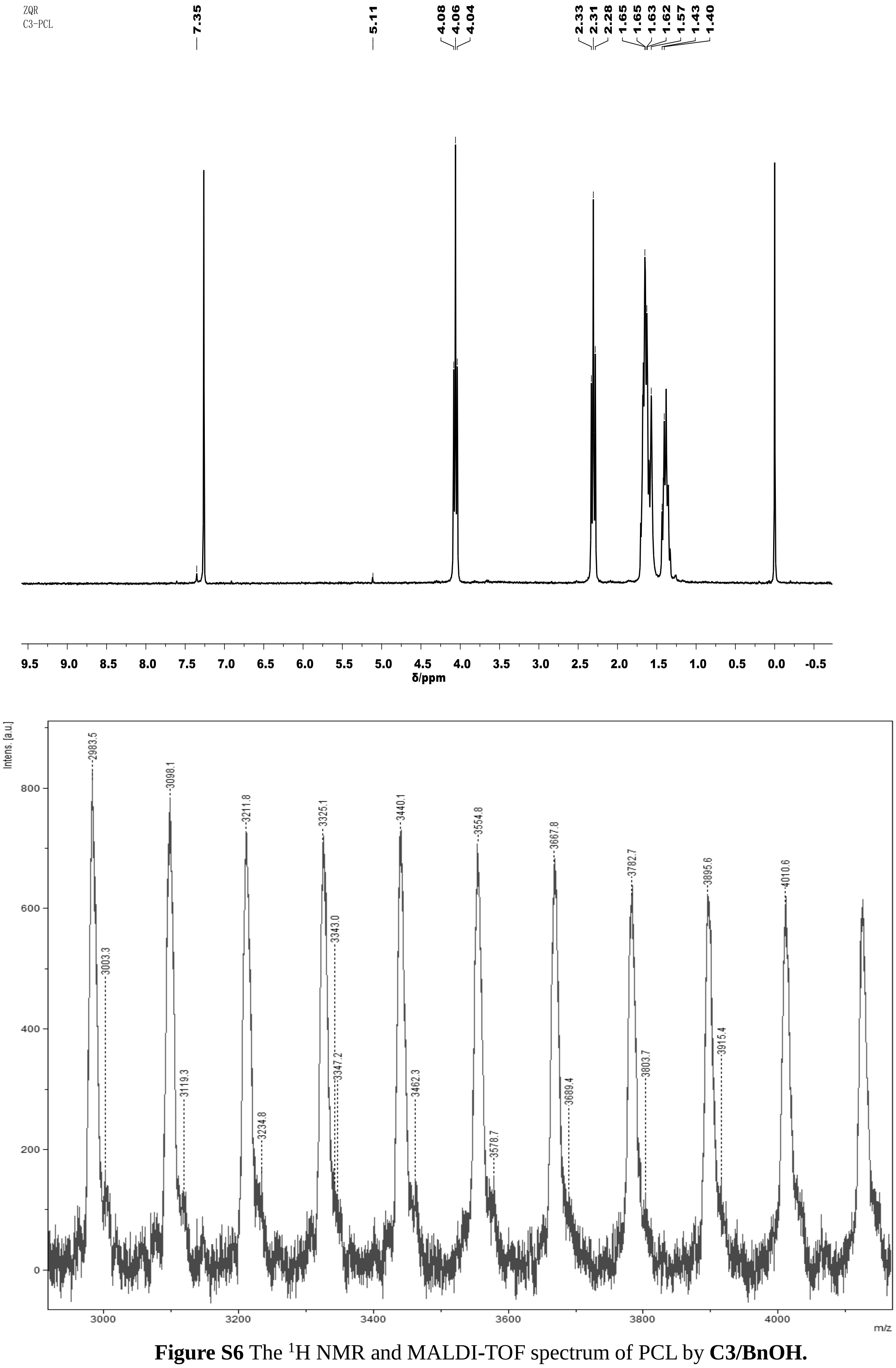

Figure S6 The ^{1}H NMR and MALDI-TOF spectrum of PCL by **C3/BnOH.**

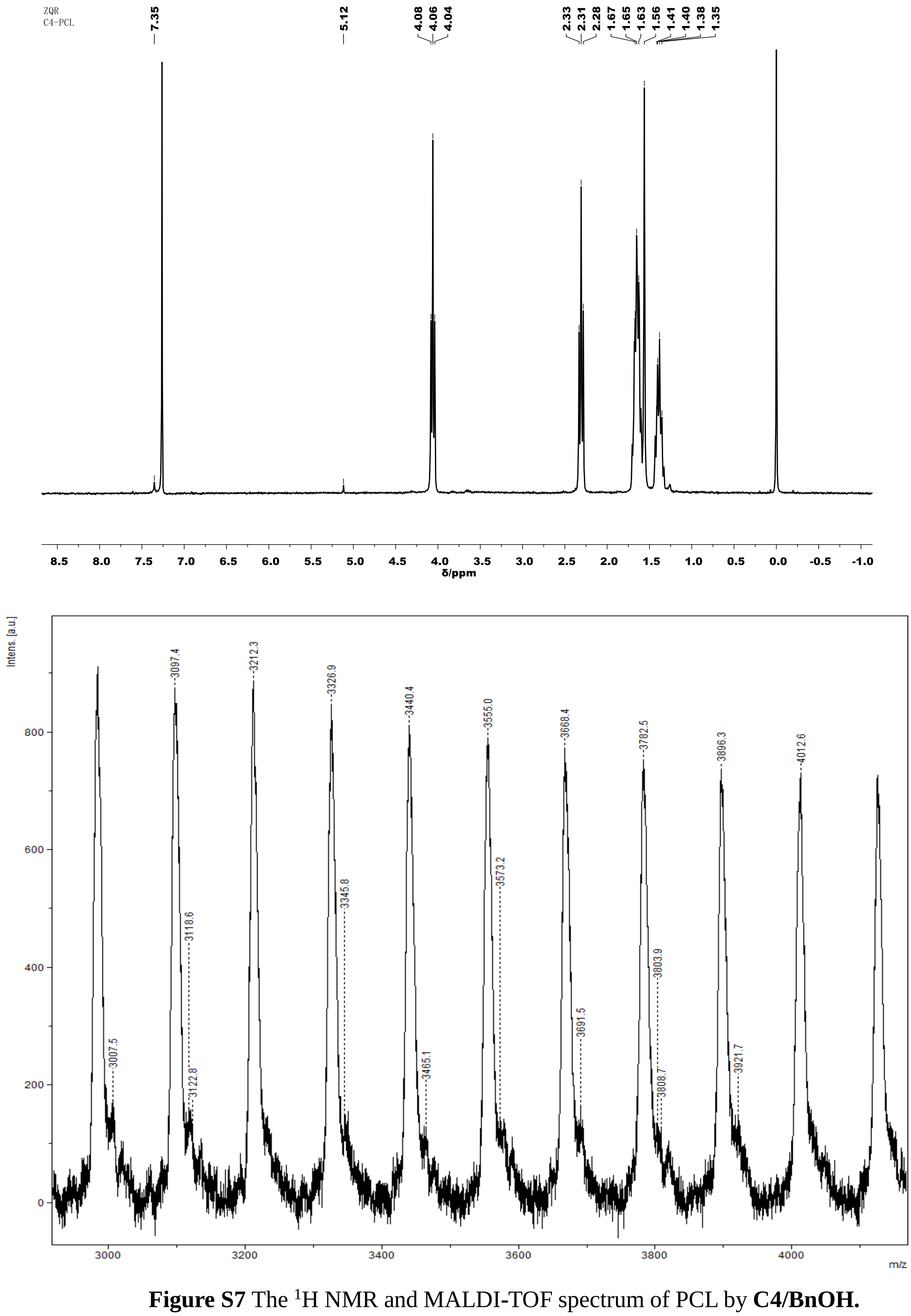

Figure S7 The ^{1}H NMR and MALDI-TOF spectrum of PCL by **C4/BnOH.**

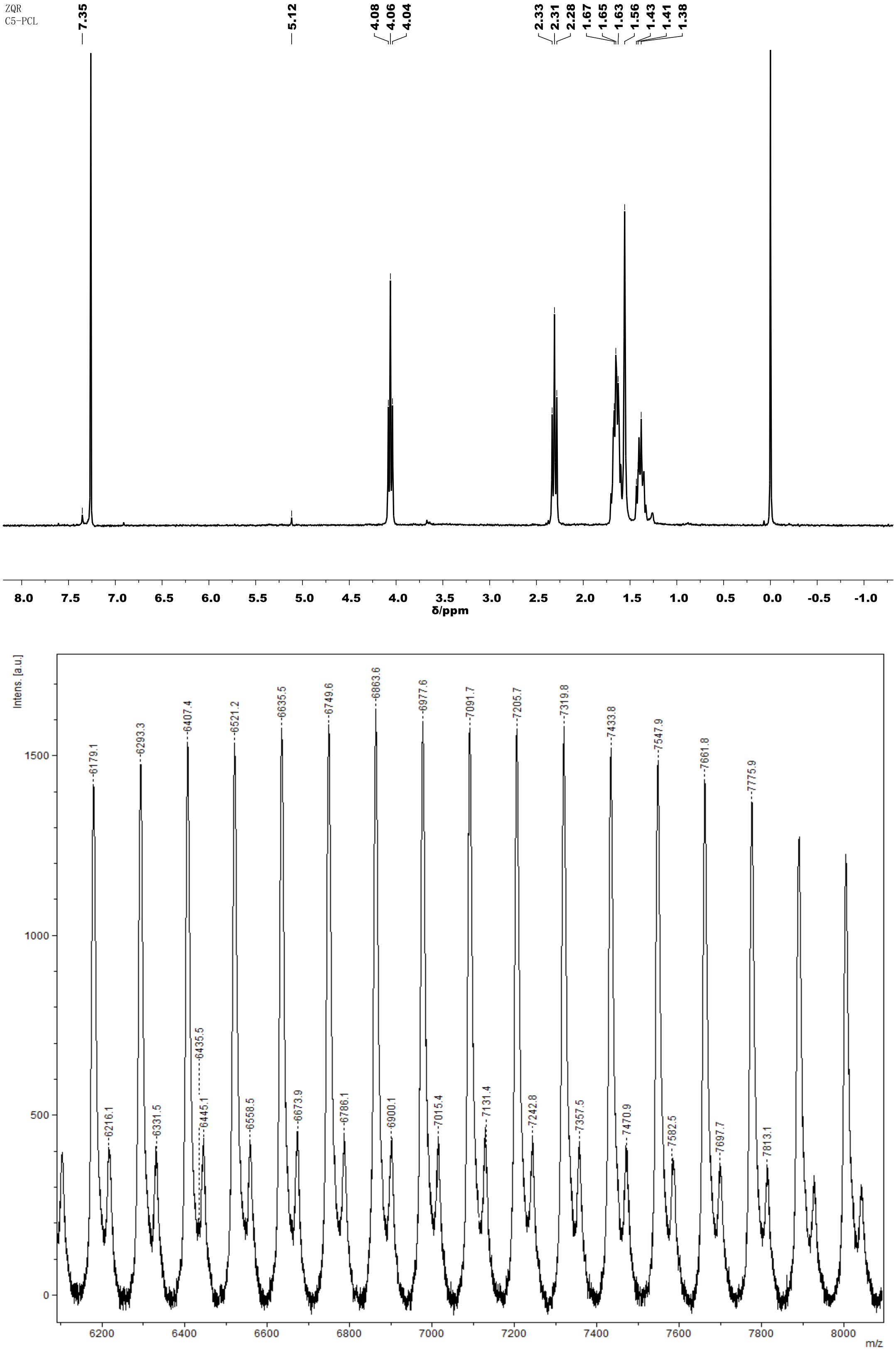

Figure S8 The ^{1}H NMR and MALDI-TOF spectrum of PCL by **C5/BnOH.**

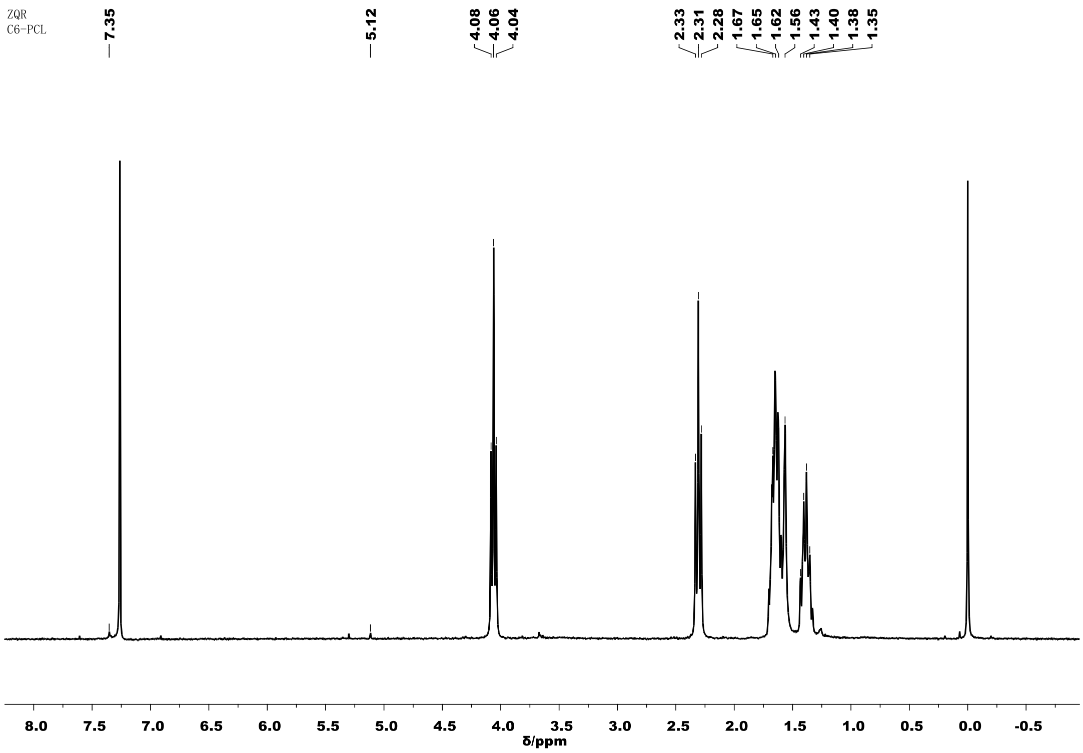

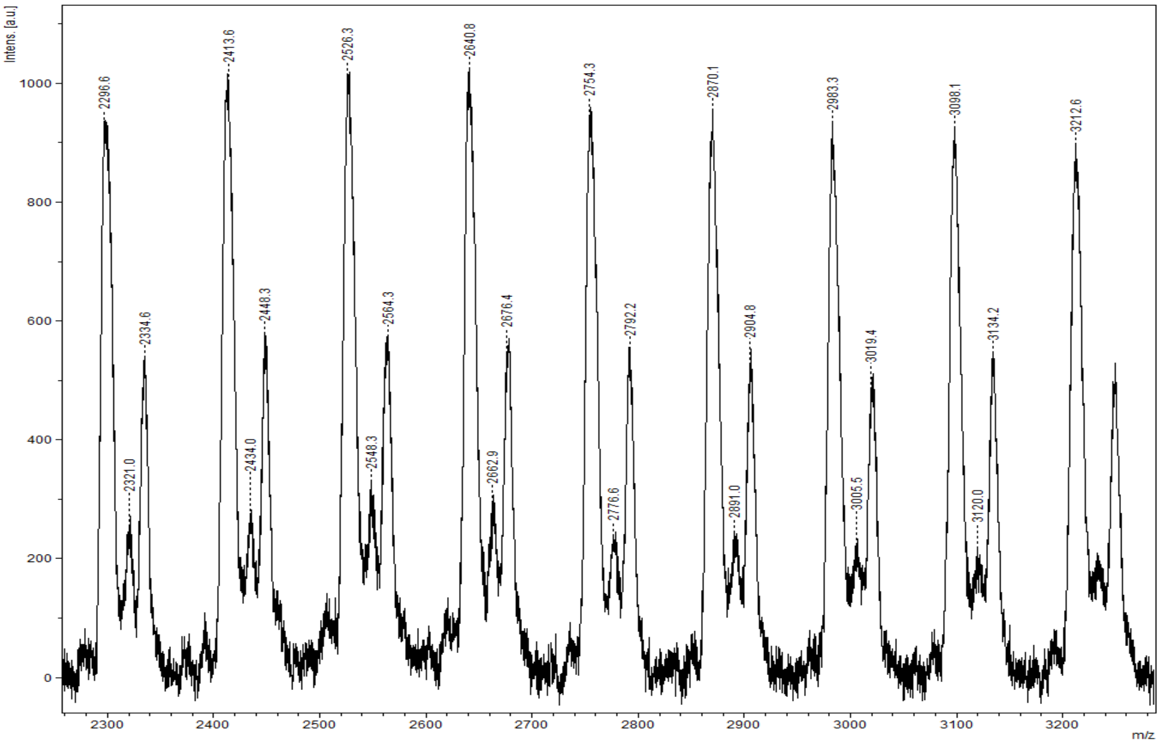

Figure S9 The ^{1}H NMR and MALDI-TOF spectrum of PCL by **C6/BnOH.**
